# Supplementary figures and images for: Palmitoylation regulates neuropilin-2 localization and function in cortical neurons and conveys specificity to semaphorin signaling via palmitoyl acyltransferases
Source: eLife. 2023 Apr 3;12:e83217. doi: 10.7554/eLife.83217 (PMC10069869; doi:10.7554/eLife.83217)

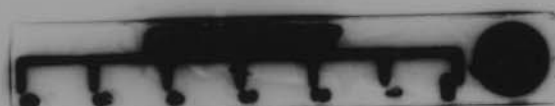

5-HABE 7-11-11  
 Exposure: ECL Plus 1'

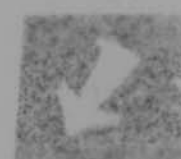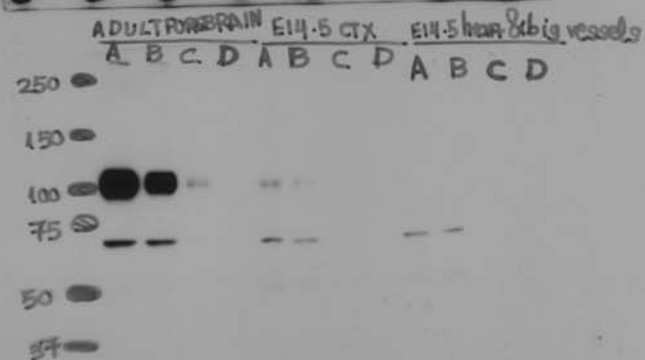

SAP102.1B

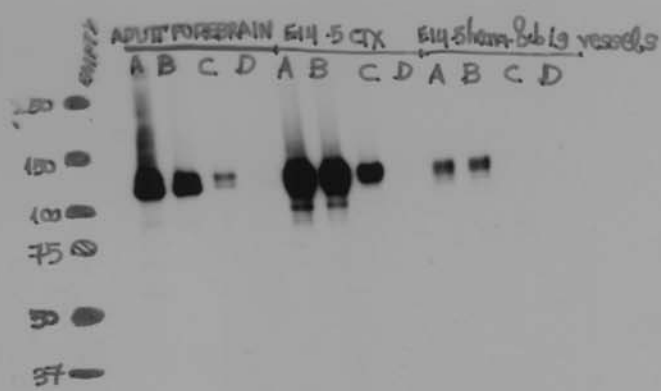

Nrp2.1B

16 µl sample / lane

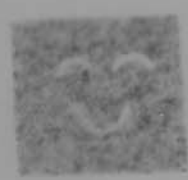

Supplement: Figure 2—source data 1. [file elife-83217-fig2-data1.pdf]

7-11-11

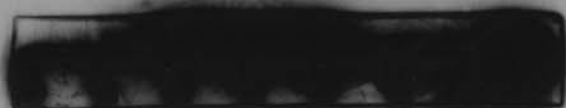

5th ABE  
ECL 14'

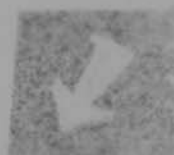

250  
150  
100  
75

SAP102 IB

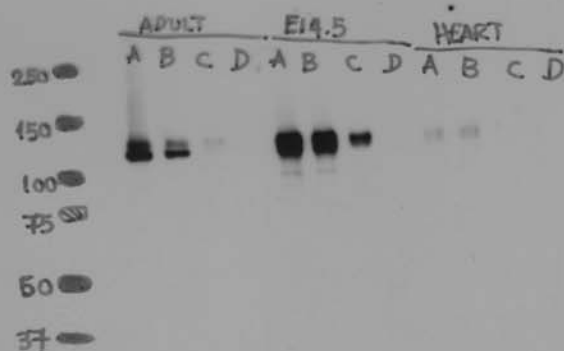

Nrp2 IB

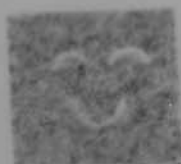

Supplement: Figure 2—source data 3. [file elife-83217-fig2-data3.pdf]

7-11-11

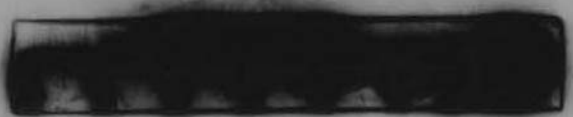

5th ABE  
ECL 14'

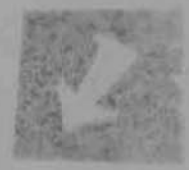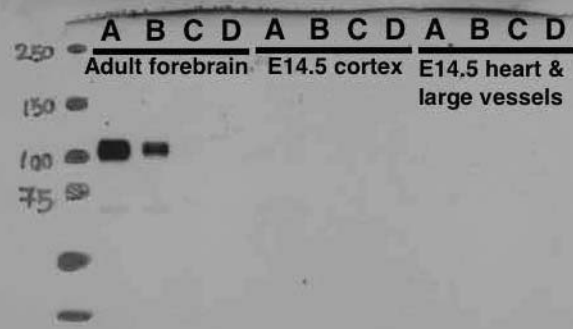

ABE on mouse tissue

SAP102 immunoblot  
SAP102 IB

A: Input +HA  
B: Input -HA  
C: +HA  
D: -HA

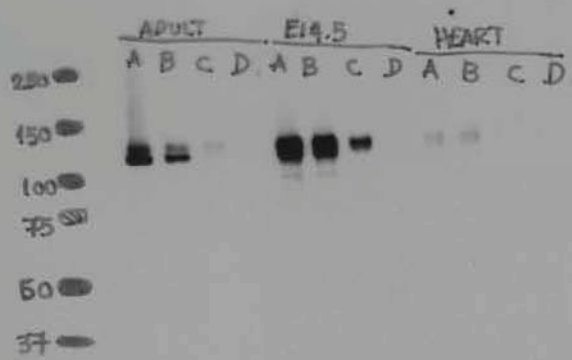

Nrp2 IB

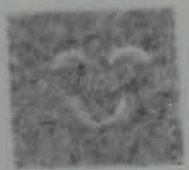

Supplement: Figure 2—source data 6. [file elife-83217-fig2-data6.pdf]

EK/

# Neuropilin-2 Immunoblots (Cell Signaling #3366S) 46th ABE

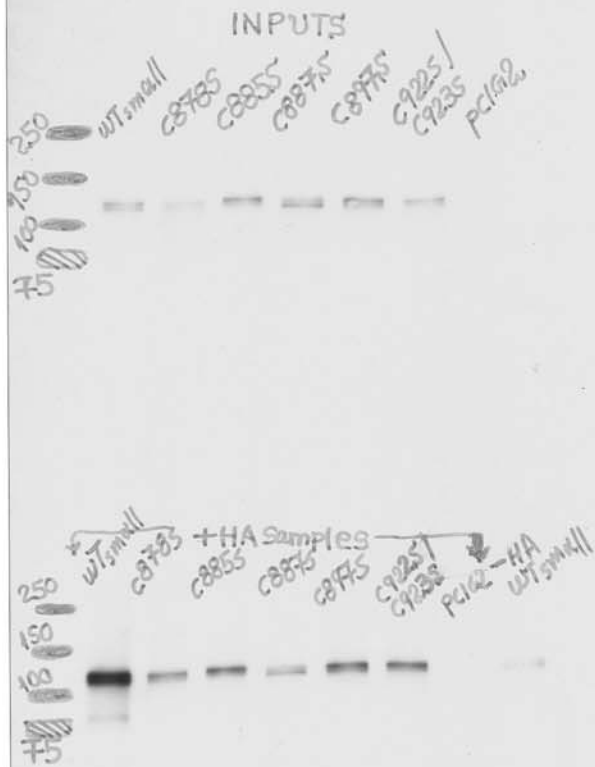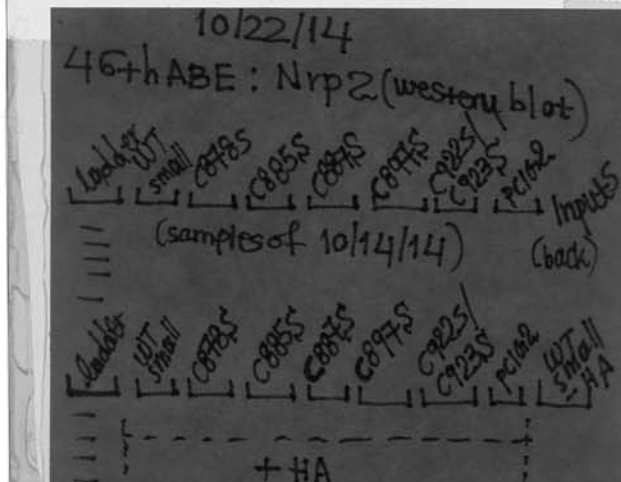

Supplement: Figure 2—source data 15. [file elife-83217-fig2-data15.pdf]

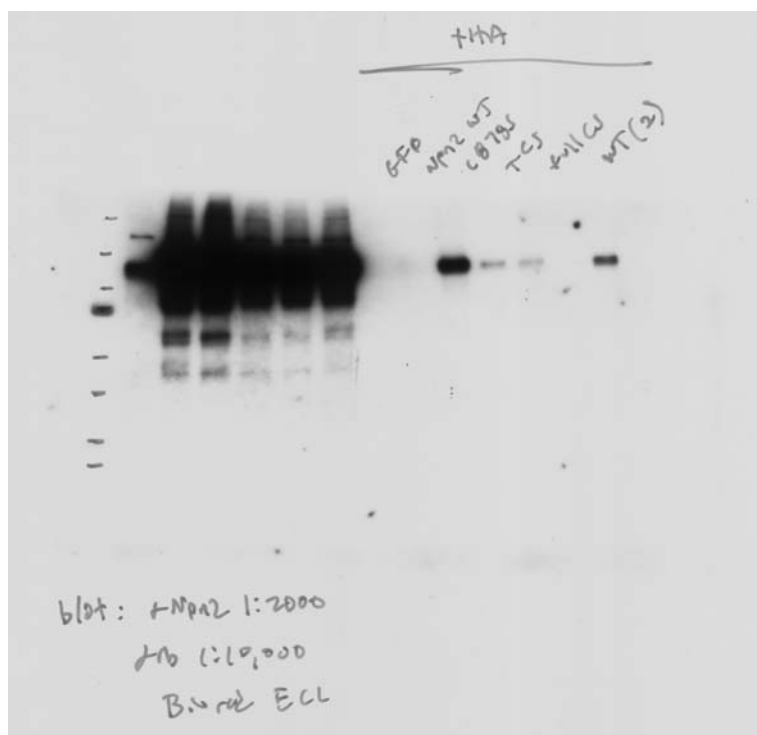

Supplement: Figure 2—source data 18. [file elife-83217-fig2-data18.pdf]

4/4/16  $\frac{2}{\text{sec.}}$

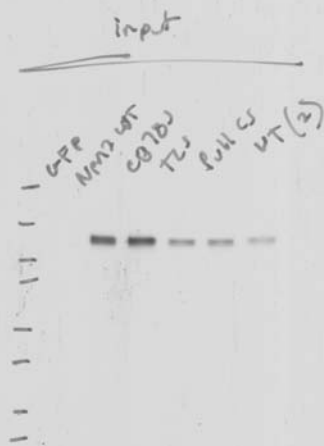

blot:  $\alpha$  NM2

Supplement: Figure 2—source data 19. [file elife-83217-fig2-data19.pdf]

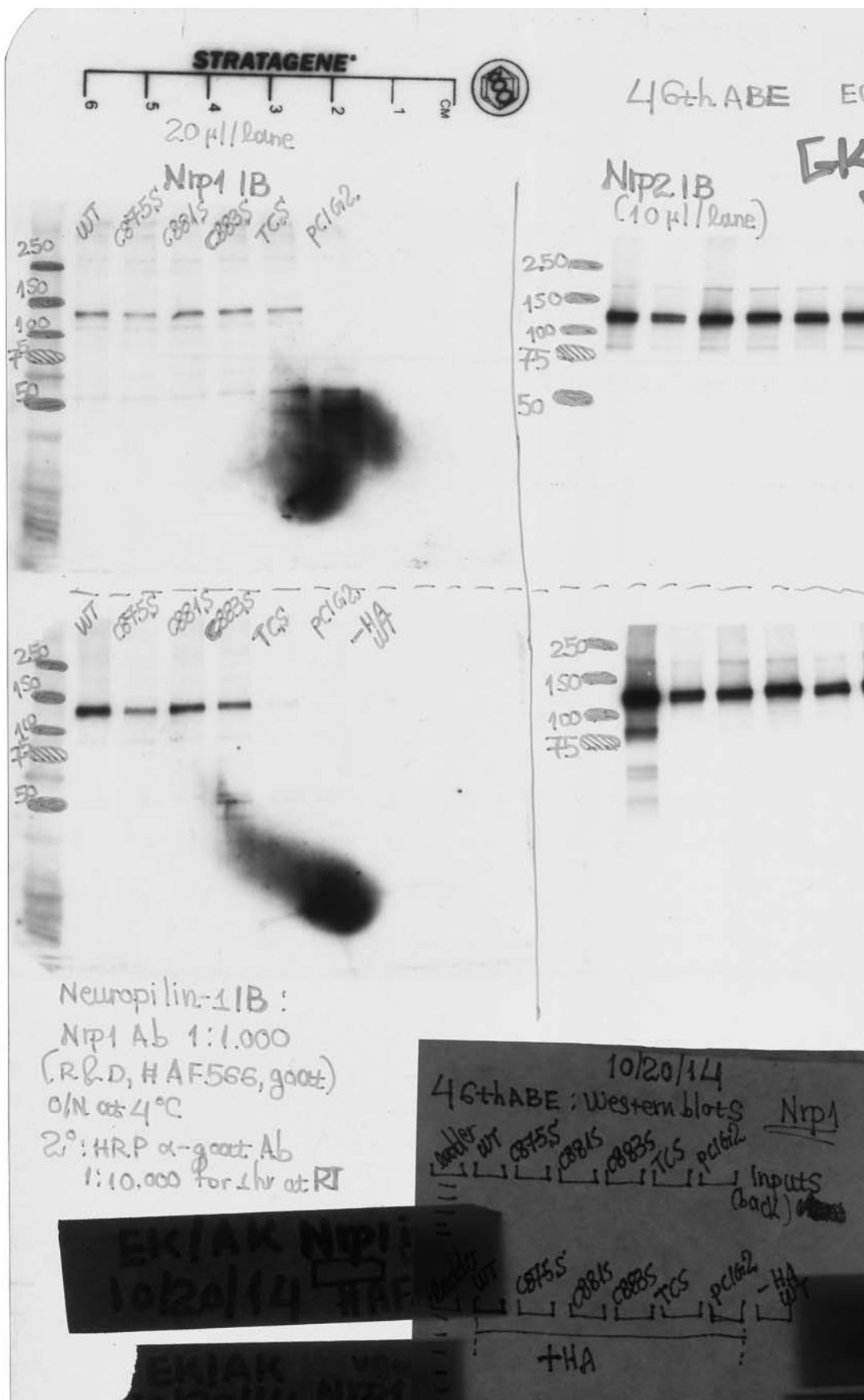

Supplement: Figure 2—source data 23. [file elife-83217-fig2-data23.pdf]

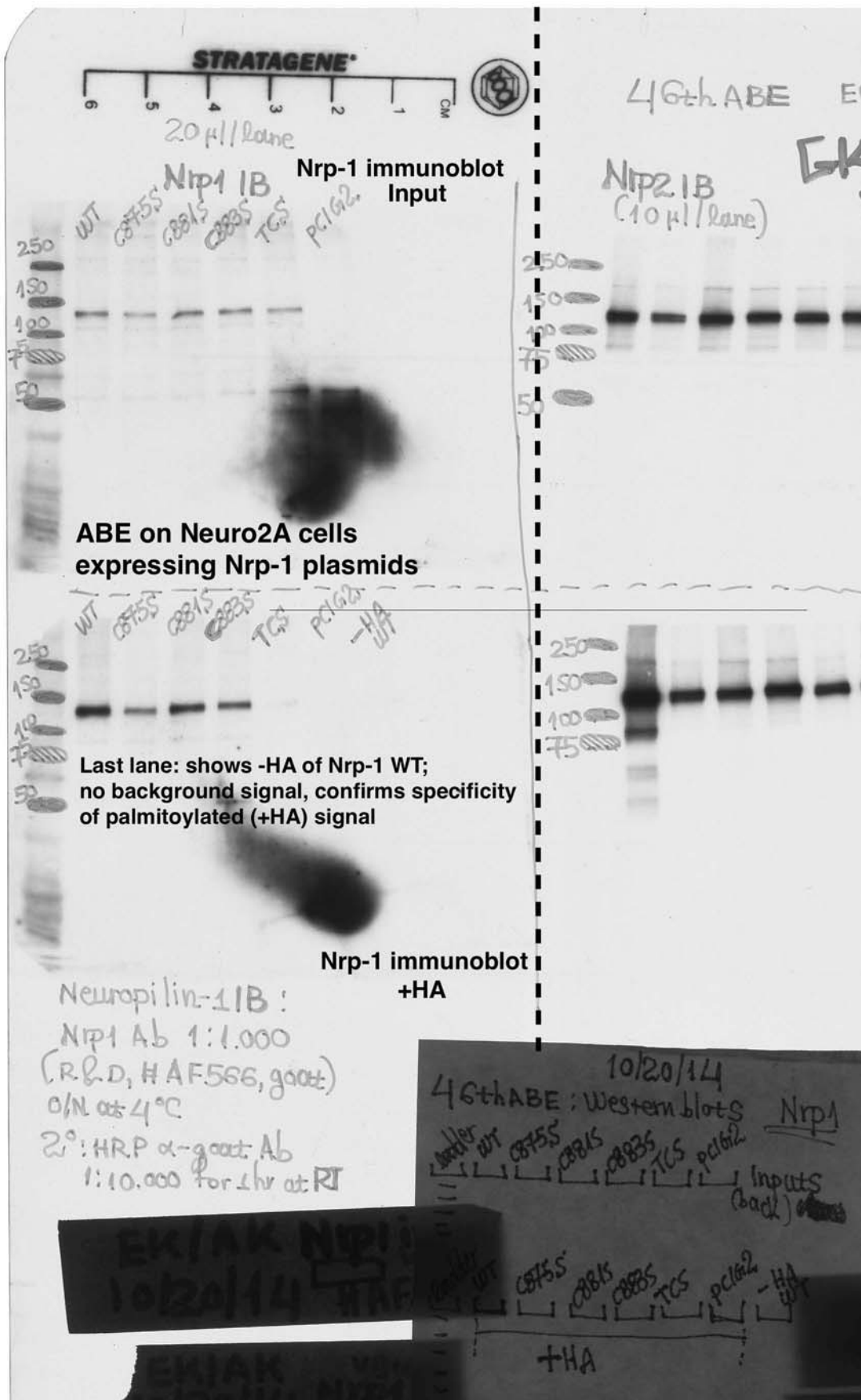

Supplement: Figure 2—source data 24. [file elife-83217-fig2-data24.pdf]

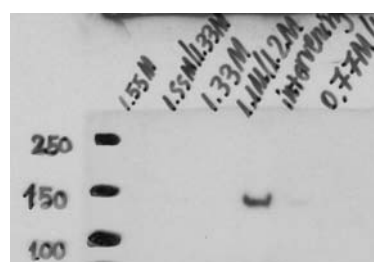

Supplement: Figure 3—figure supplement 3—source data 1. [file elife-83217-fig3-figsupp3-data1.pdf]

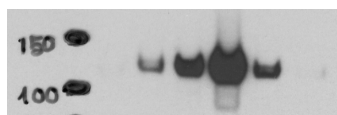

Supplement: Figure 3—figure supplement 3—source data 2. [file elife-83217-fig3-figsupp3-data2.pdf]

## Golgi isolation from mouse whole brain

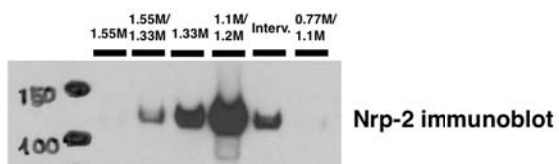

Supplement: Figure 3—figure supplement 3—source data 4. [file elife-83217-fig3-figsupp3-data4.pdf]

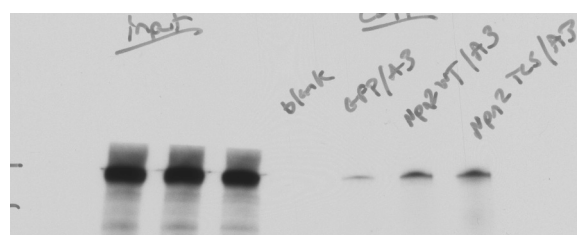

Supplement: Figure 3—figure supplement 4—source data 1. [file elife-83217-fig3-figsupp4-data1.pdf]

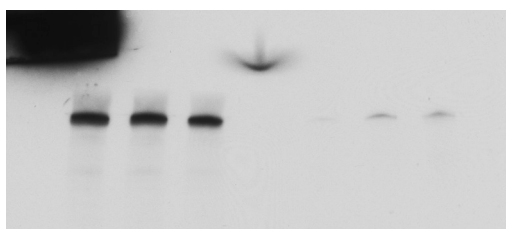

Supplement: Figure 3—figure supplement 4—source data 2. [file elife-83217-fig3-figsupp4-data2.pdf]

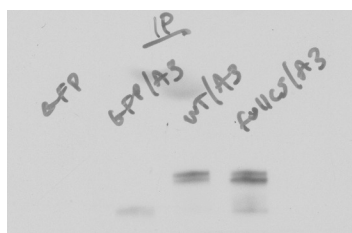

Supplement: Figure 3—figure supplement 4—source data 3. [file elife-83217-fig3-figsupp4-data3.pdf]

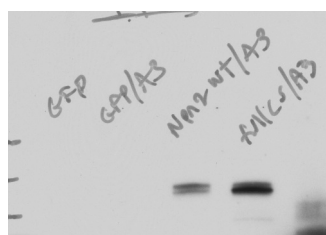

Supplement: Figure 3—figure supplement 4—source data 4. [file elife-83217-fig3-figsupp4-data4.pdf]

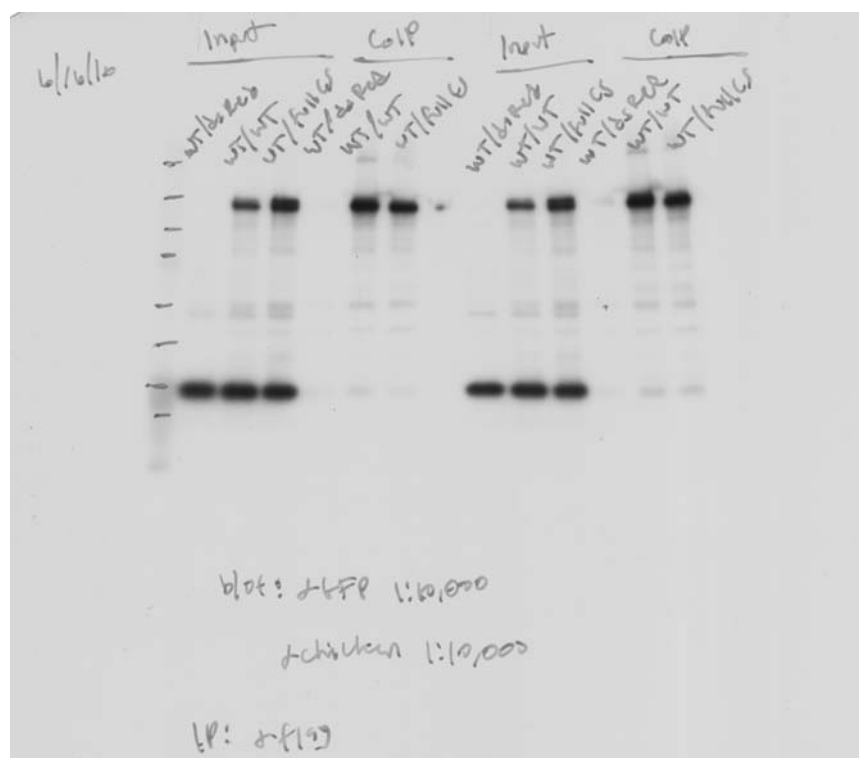

Supplement: Figure 3—figure supplement 4—source data 10. [file elife-83217-fig3-figsupp4-data10.pdf]

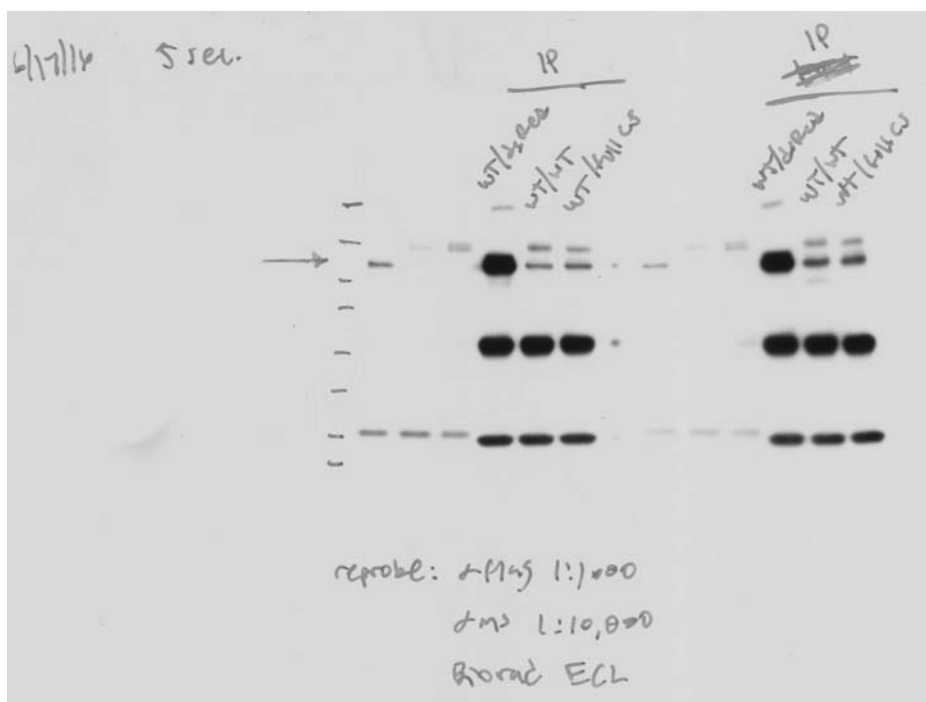

Supplement: Figure 3—figure supplement 4—source data 11. [file elife-83217-fig3-figsupp4-data11.pdf]

repeat 21

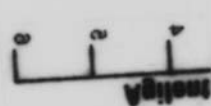

42

WT/WT  
WT/WT  
WT/WT

wt/wt  
wt/wt  
wt/wt

blot: flag 1:600

time 1: 60,000

Biorak ECL - 5 sec.

Supplement: Figure 3—figure supplement 4—source data 12. [file elife-83217-fig3-figsupp4-data12.pdf]

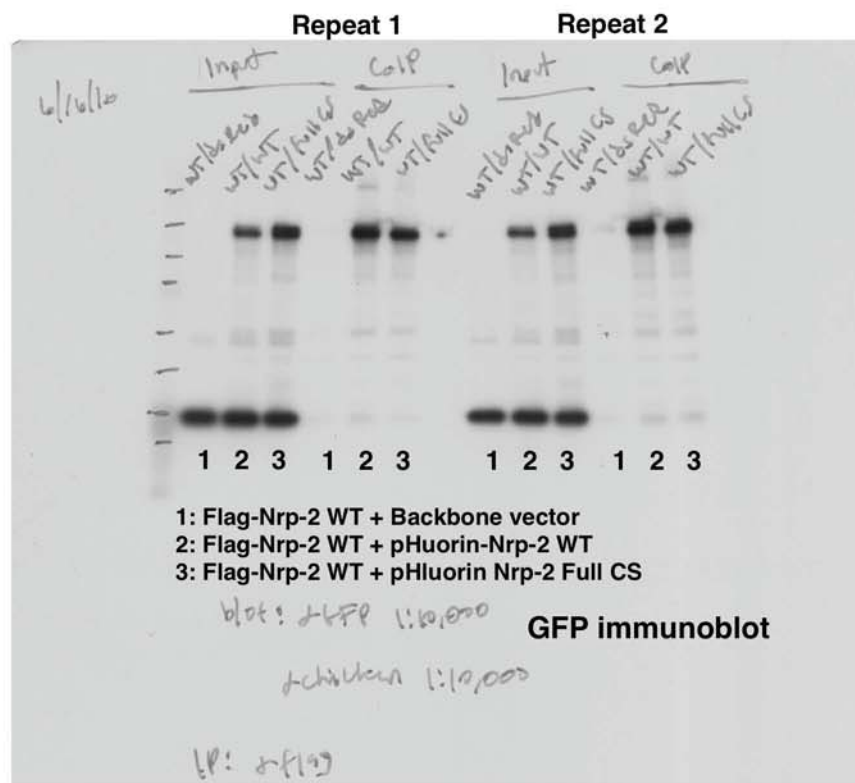

Supplement: Figure 3—figure supplement 4—source data 13. [file elife-83217-fig3-figsupp4-data13.pdf]

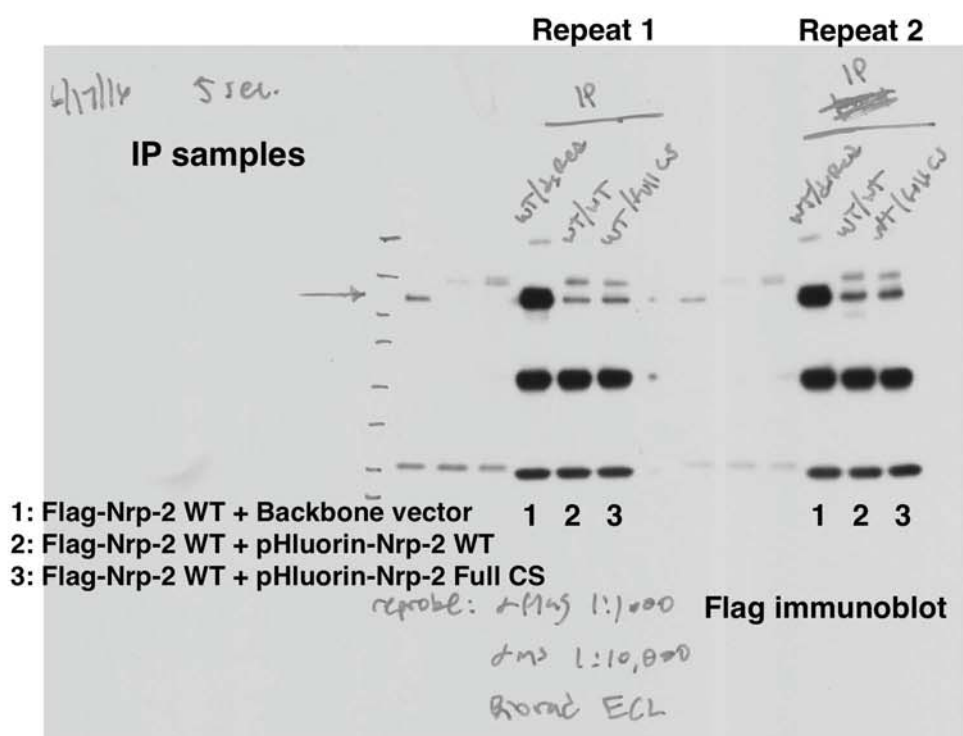

Supplement: Figure 3—figure supplement 4—source data 14. [file elife-83217-fig3-figsupp4-data14.pdf]

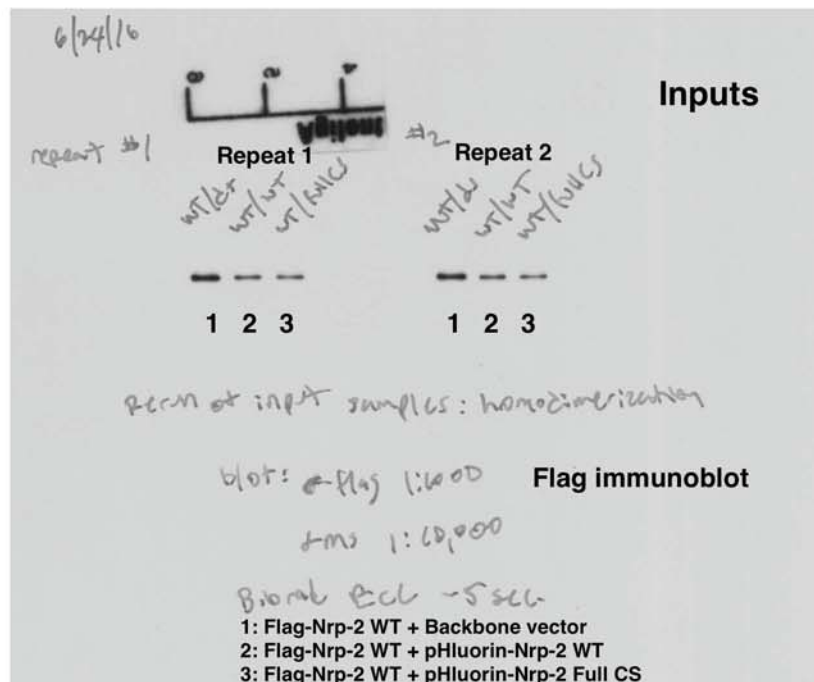

Supplement: Figure 3—figure supplement 4—source data 15. [file elife-83217-fig3-figsupp4-data15.pdf]

7/13/16 25cc.

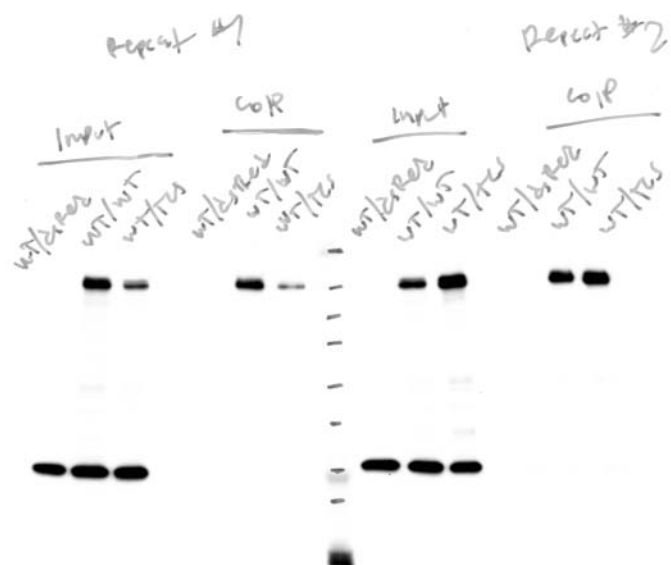

blot! 26kDa 1:10,000  
 110kDa 1:10,000  
 BioRad ECL

Supplement: Figure 3—figure supplement 4—source data 17. [file elife-83217-fig3-figsupp4-data17.pdf]

7/14/16 bel.

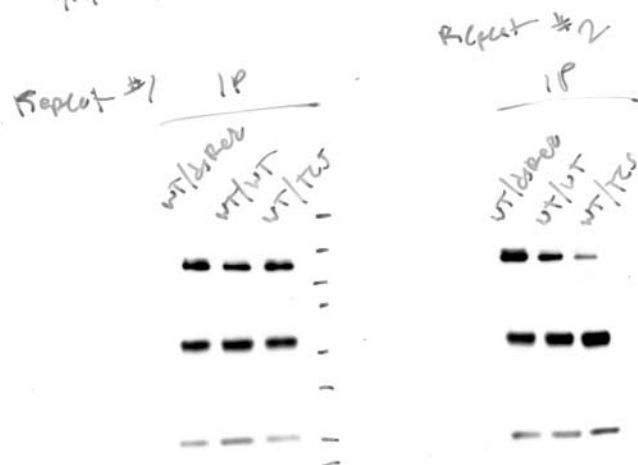

probe: flag 1:600  
 2nd 1:10,000  
 Birac E66

Supplement: Figure 3—figure supplement 4—source data 18. [file elife-83217-fig3-figsupp4-data18.pdf]

7/14/14 1hr.

Repeat #1

Input

WT/Control  
WT/WT  
WT/TG

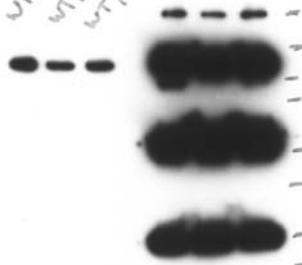

Repeat #2

Input

WT/Control  
WT/WT  
WT/TG

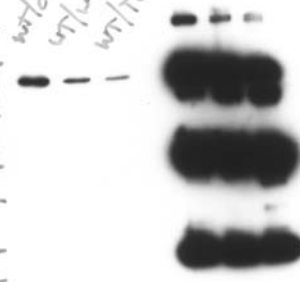

reprobe: 2Flag

Supplement: Figure 3—figure supplement 4—source data 19. [file elife-83217-fig3-figsupp4-data19.pdf]

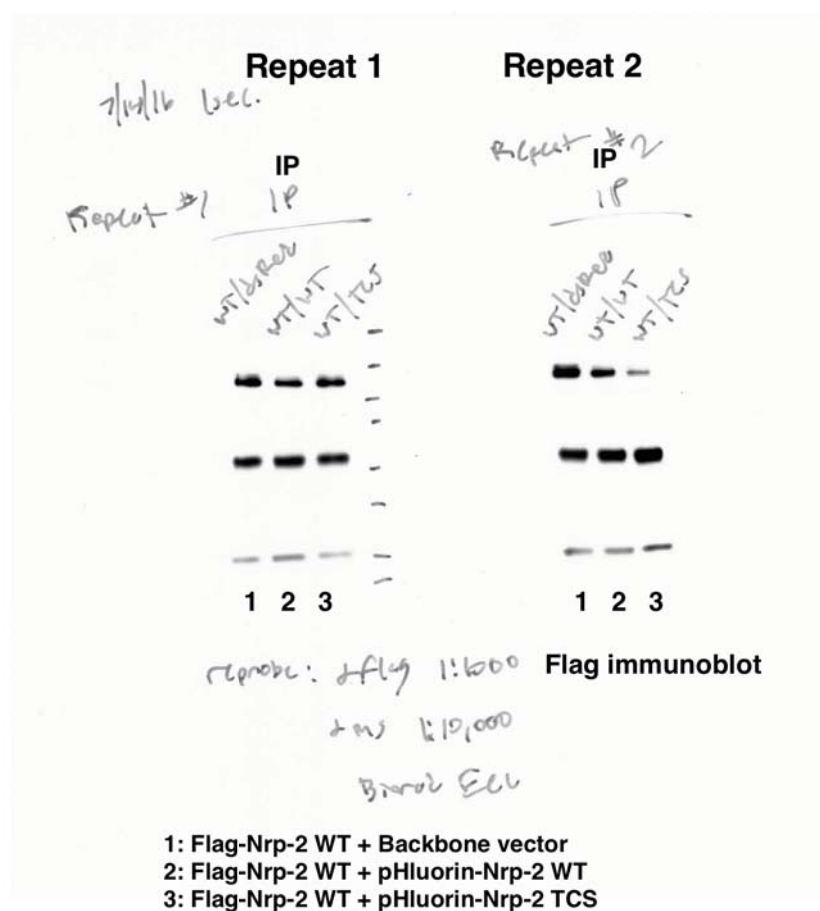

Supplement: Figure 3—figure supplement 4—source data 21. [file elife-83217-fig3-figsupp4-data21.pdf]

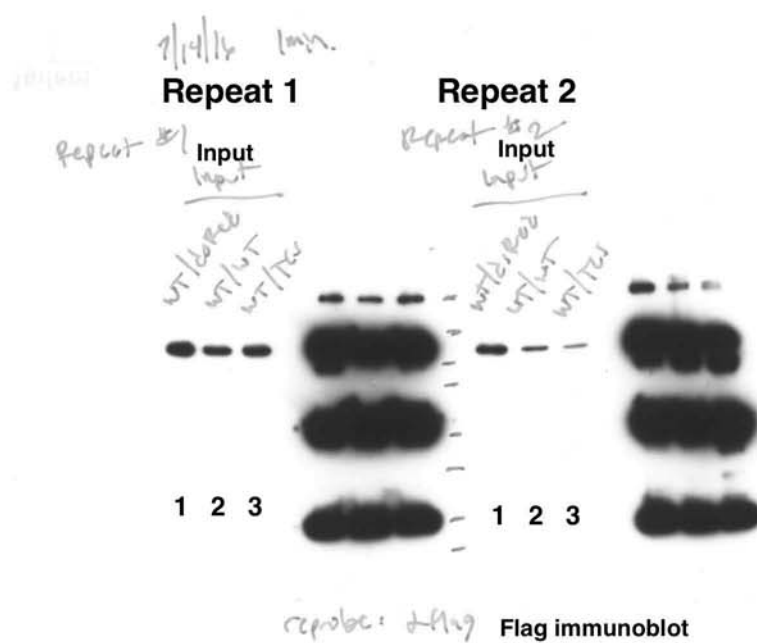

- 1: Flag-Nrp-2 WT + Backbone vector
- 2: Flag-Nrp-2 WT + pHluorin-Nrp-2 WT
- 3: Flag-Nrp-2 WT + pHluorin-Nrp-2 TCS

Supplement: Figure 3—figure supplement 4—source data 22. [file elife-83217-fig3-figsupp4-data22.pdf]

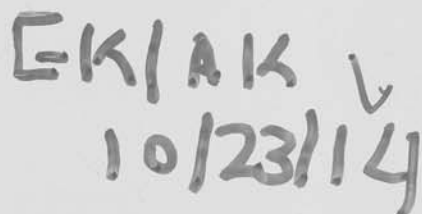

10  $\mu$ l/lane (all samples)

45th ABE

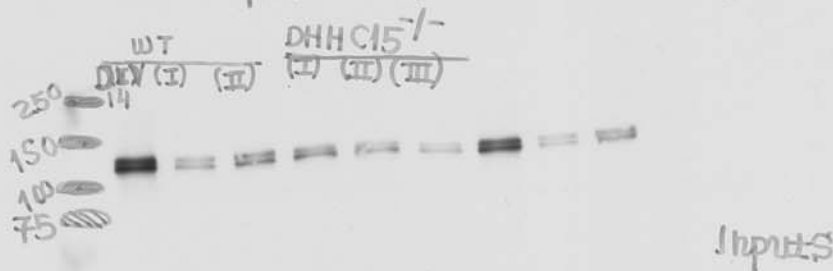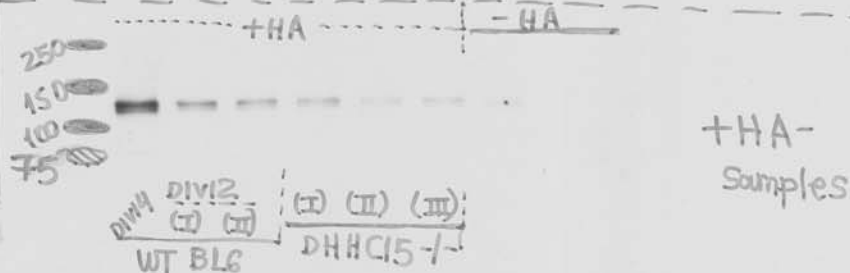

Neuropilin-2 IB:  
Nrp2 Ab 1:1,000  
(Cell Sign. #3366S)  
O/N at 4 °C  
2°: HRP-conjug.  
α-rabbit Ab  
1:10,000  
1 hr at RT

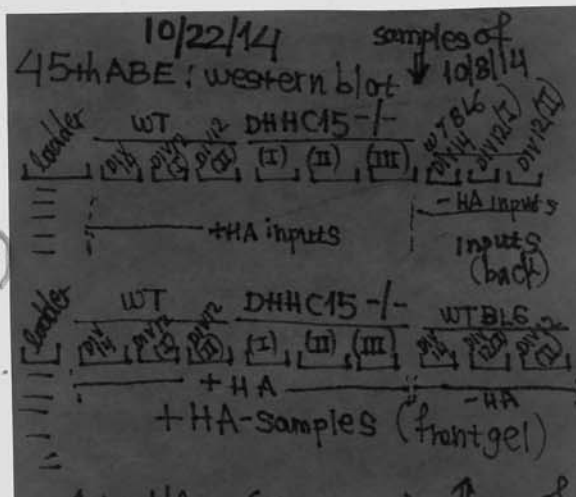

Supplement: Figure 6—source data 1. [file elife-83217-fig6-data1.pdf]
