# Supplementary material for: Palmitoylation regulates neuropilin-2 localization and function in cortical neurons and conveys specificity to semaphorin signaling via palmitoyl acyltransferases
Source: eLife. 2023 Apr 3;12:e83217. doi: 10.7554/eLife.83217 (PMC10069869; doi:10.7554/eLife.83217)
Supplement: Figure 2—source data 4. [file elife-83217-fig2-data4.pdf]

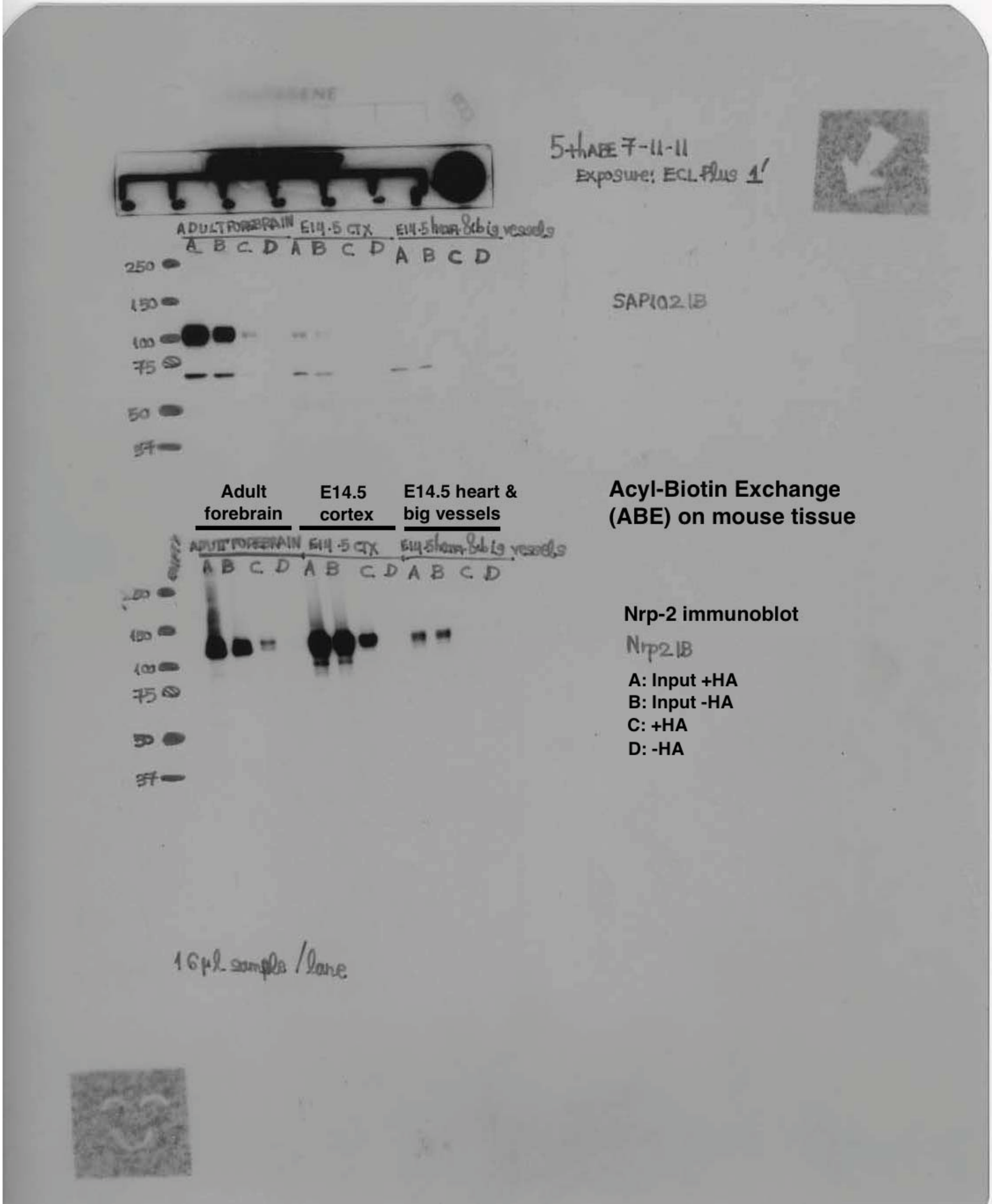
5th ABE 7-11-11
Exposure: ECL Plus 1'
ADULT FOREBRAIN E14.5 CTX E14.5 heart & big vessels
A B C D A B C D A B C D
250
150
100
75
50
37
SAP102 IB
Adult forebrain
E14.5 cortex
E14.5 heart & big vessels
Acyl-Biotin Exchange (ABE) on mouse tissue
Nrp-2 immunoblot
Nrp2 IB
A: Input +HA
B: Input -HA
C: +HA
D: -HA
16 µl sample/lane
