# Supplementary material for: Palmitoylation regulates neuropilin-2 localization and function in cortical neurons and conveys specificity to semaphorin signaling via palmitoyl acyltransferases
Source: eLife. 2023 Apr 3;12:e83217. doi: 10.7554/eLife.83217 (PMC10069869; doi:10.7554/eLife.83217)
Supplement: Figure 2—source data 5. [file elife-83217-fig2-data5.pdf]

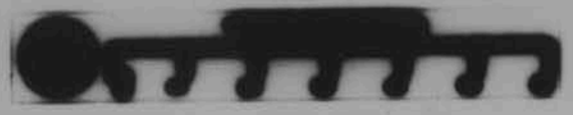

7-7-11 5th ABE Exposure: ECL 5'

Acyl-Biotin Exchange IN VIVO from adult mouse forebrain, E14.5 mouse ~~[illegible]~~ cortex and E14.5 heart and big vessels.

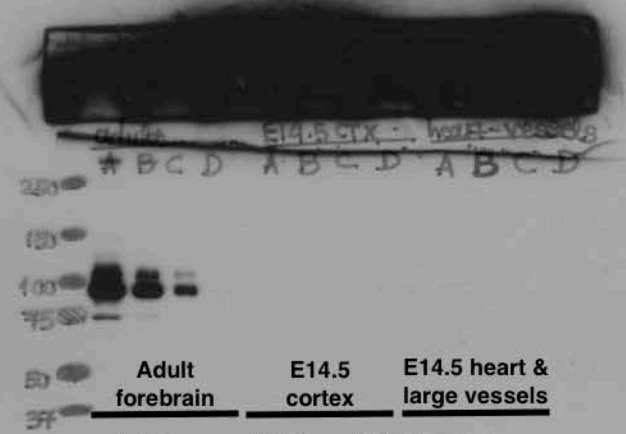

ABE on mouse tissue

PSD-95 immunoblot

PSD-95 IB: α-PSD-95 Ab, mouse, Millipore

blocking: 5% milk, 1hr at RT

1° Ab: 1:2000 in 5% milk/TBS-T o/N, 4°C

2° Ab: 1:10000 in 1% milk, 1hr, RT

A: Input +HA **B: Input -HA**

C: +HA **D: -HA**

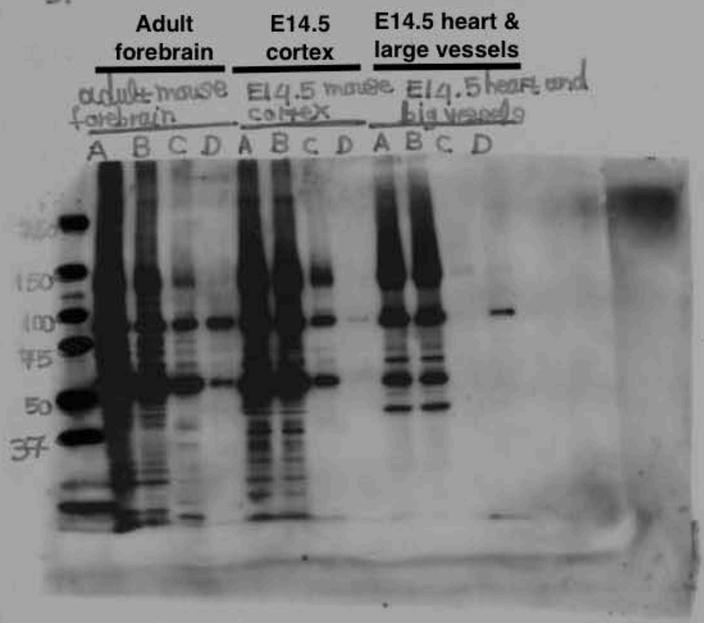

Nrp-1 immunoblot

Nrp1 IB: α-Nrp1 Ab, goat (R&D)

blocking: 3% BSA in TBS-T for 1hr at RT

1° Ab: 1:1000 in 3% BSA/TBS-T o/N at 4°C

2° Ab: α-goat HRP-conjugated 2° Ab

1:10.000 in 1% milk, 1hr at RT
