# Supplementary material for: Palmitoylation regulates neuropilin-2 localization and function in cortical neurons and conveys specificity to semaphorin signaling via palmitoyl acyltransferases
Source: eLife. 2023 Apr 3;12:e83217. doi: 10.7554/eLife.83217 (PMC10069869; doi:10.7554/eLife.83217)
Supplement: Figure 2—source data 7. [file elife-83217-fig2-data7.pdf]

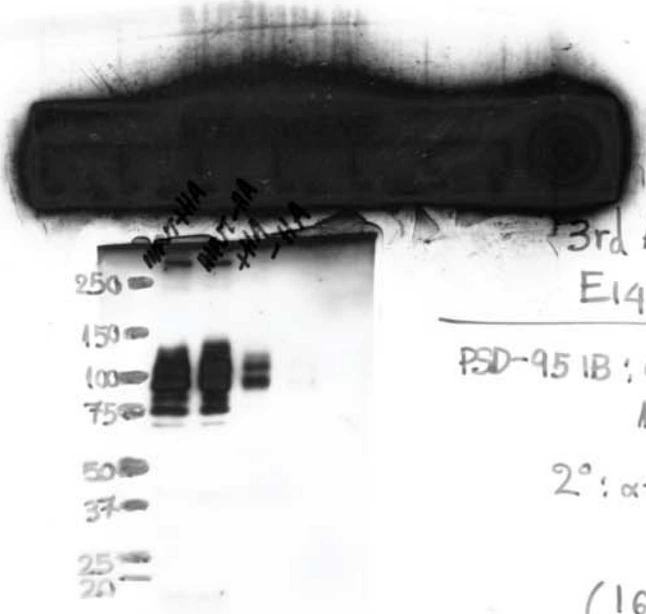

3rd ABE 5-7-11 Exposure ECL 10'

3rd Acyl-Biotin Exchange from
E14.5 DIV28 cortical neurons (culture)

PSD-95 IB: α-PSD-95, mouse monoclonal (Millipore)
1:2.000

2°: α-mouse HRP-conjugated Ab
1:10.000 in 1% milk

(16 µl/lane) from (120µl sample + 40µl 4x Laemmli)

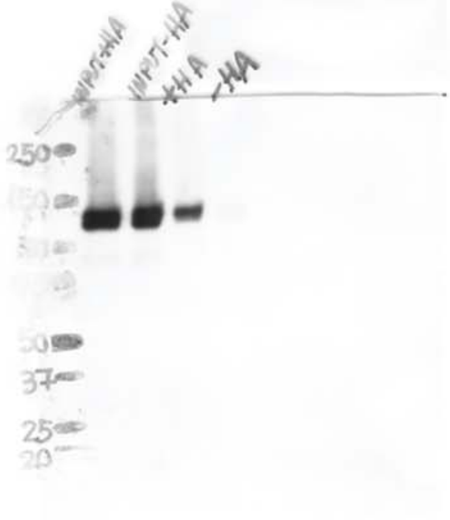

Nlp2 IB: α-Nlp2 Ab, rabbit (Cell Signaling)
1:1000 in 5% milk

2°: α-rabbit HRP-conjugated Ab
1:10.000 in 1% milk

(16 µl/lane) from (120µl sample + 40µl 4x Laemmli)
