# Supplementary material for: Palmitoylation regulates neuropilin-2 localization and function in cortical neurons and conveys specificity to semaphorin signaling via palmitoyl acyltransferases
Source: eLife. 2023 Apr 3;12:e83217. doi: 10.7554/eLife.83217 (PMC10069869; doi:10.7554/eLife.83217)
Supplement: Figure 2—source data 8. [file elife-83217-fig2-data8.pdf]

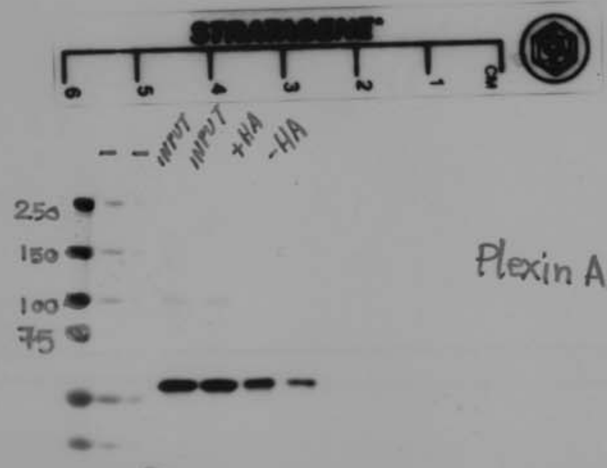

3rd ABE 5-12-11
Exposure: ECL 30"

Plexin A3 Ab, rabbit (AbCam)

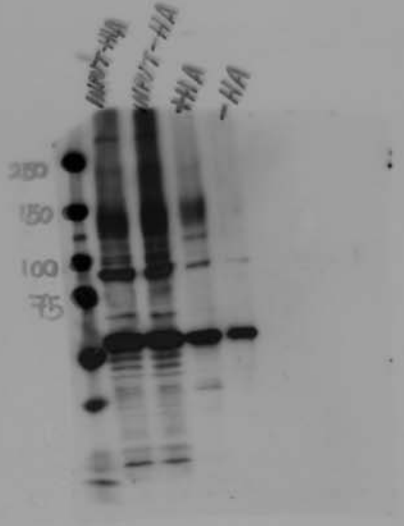

Nrp1 IB: α-Nrp1 Ab, goat (RandD)
1:1000 in 3% BSA/TBS-T overnight at 4°C

2°: α-goat HRP-conjugated Ab
1:10,000 in 1% milk

16 μl/lane from [120 μl sample + 40 μl 4X Laemmli]
