# Supplementary material for: Palmitoylation regulates neuropilin-2 localization and function in cortical neurons and conveys specificity to semaphorin signaling via palmitoyl acyltransferases
Source: eLife. 2023 Apr 3;12:e83217. doi: 10.7554/eLife.83217 (PMC10069869; doi:10.7554/eLife.83217)
Supplement: Figure 2—source data 9. [file elife-83217-fig2-data9.pdf]

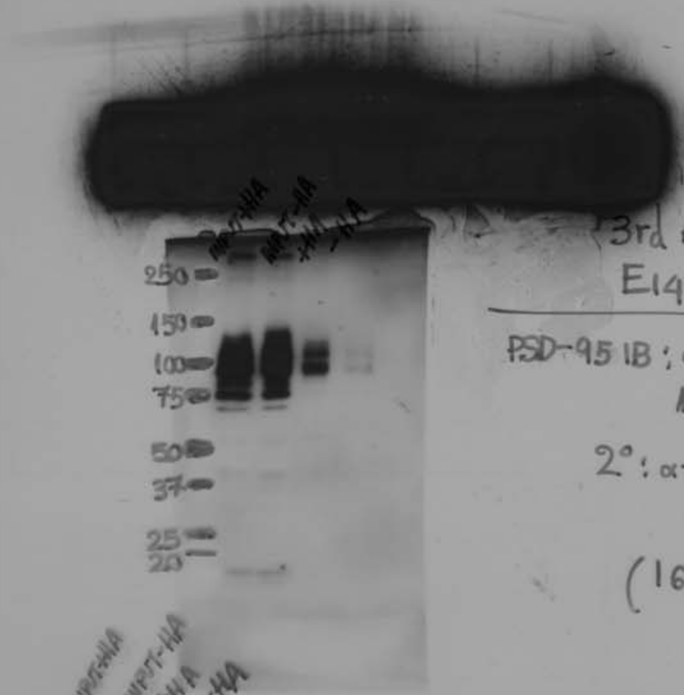

3rd ABE 5-7-11 Exposure ECL 10'

3rd Acyl-Biotin Exchange from E14.5 DIV28 cortical neurons (culture)

PSD-95 IB : α-PSD-95, mouse monoclonal (Millipore)
1:2000

2°: α-mouse HRP-conjugated Ab
1:10.000 in 1% milk

(16 μl/lane) from (120μl sample + 40μl 4x Laemmli)

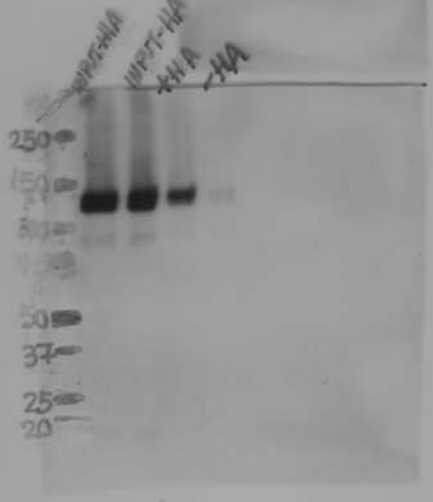

Nip2 IB: α-Nip2 Ab, rabbit (Cell Signalling)
1:1000 in 5% milk

2°: α-rabbit HRP-conjugated Ab
1:10.000 in 1% milk

(16 μl/lane) from (120μl sample + 40μl 4x Laemmli)
