# Supplementary material for: Palmitoylation regulates neuropilin-2 localization and function in cortical neurons and conveys specificity to semaphorin signaling via palmitoyl acyltransferases
Source: eLife. 2023 Apr 3;12:e83217. doi: 10.7554/eLife.83217 (PMC10069869; doi:10.7554/eLife.83217)
Supplement: Figure 2—source data 10. [file elife-83217-fig2-data10.pdf]

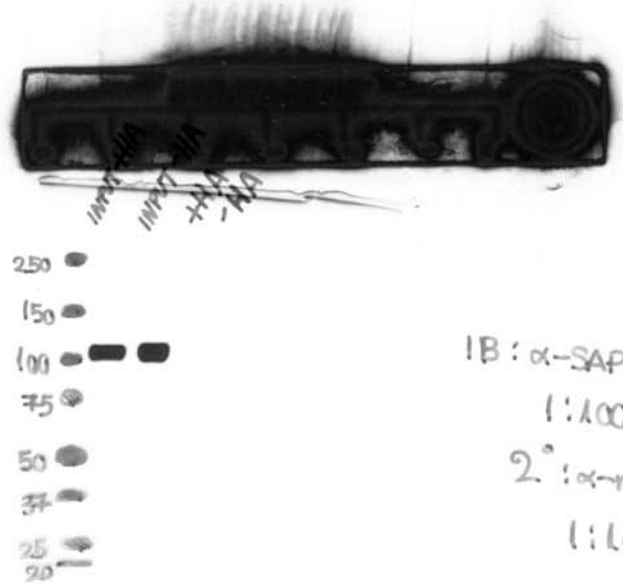

5-9-11 3rd ABE
Exposure: ECL Plus 1'

IB: α-SAP102 Ab, mouse monoclonal (NeuroMab)
1:1000 in 5% milk for 1hr at RT
2°: α-mouse HRP-conjugated
1:10000 in 1% milk

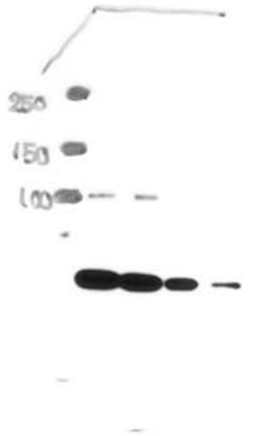

IB: α-Plexin A3 Ab
did not work because I blocked in milk instead of BSA.

3rd ABE (Acyl-Biotin Exchange) from Primary cortical neurons (culture)
E14.5 DIV28

16 μl/lane from [120μl sample + 40μl 4x Laemmli buffer]
