# Supplementary material for: Palmitoylation regulates neuropilin-2 localization and function in cortical neurons and conveys specificity to semaphorin signaling via palmitoyl acyltransferases
Source: eLife. 2023 Apr 3;12:e83217. doi: 10.7554/eLife.83217 (PMC10069869; doi:10.7554/eLife.83217)
Supplement: Figure 2—source data 11. [file elife-83217-fig2-data11.pdf]

ABE on deep layer primary cortical neurons

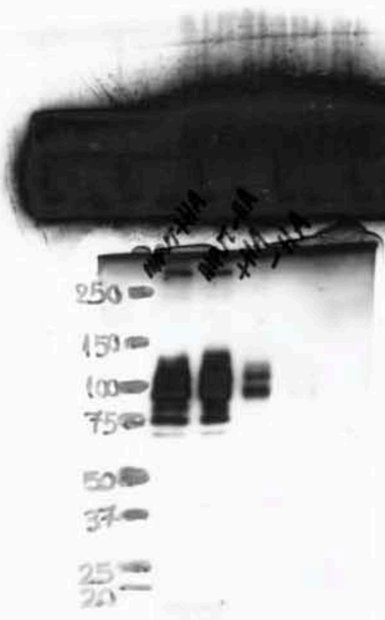

3rd ABE 5-7-11 Exposure ECL 10'

3rd Acyl-Bio[illegible]in Exchange from
E14.5 DIV28 cortical neurons (culture)

PSD-95 IB : α-PSD-95, mouse monoclonal (Millipore)
1:2000

2° : α-mouse HRP-conjugated Ab
1:10.000 in 1% milk

(16 μl/lane) from (120 μl sample + 40 μl 4x Laemmli)

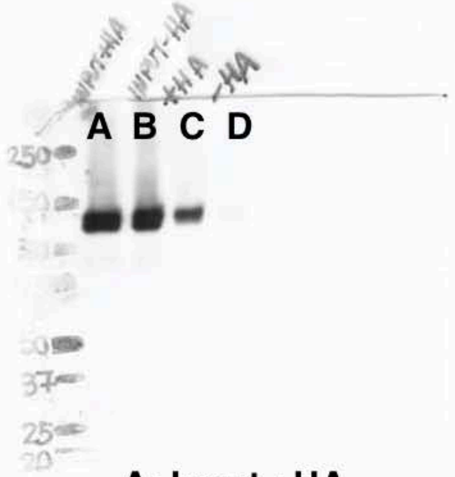

Nrp-2 immunoblot

Nrp2 IB : α-Nrp2 Ab, rabbit (Cell Signaling)
1:1000 in 5% milk

2° : α-rabbit HRP-conjugated Ab
1:10.000 in 1% milk

(16 μl/lane) from (120 μl sample + 40 μl 4x Laemmli)

A: Input +HA
B: Input -HA
C: +HA
D: -HA
