# Supplementary material for: Palmitoylation regulates neuropilin-2 localization and function in cortical neurons and conveys specificity to semaphorin signaling via palmitoyl acyltransferases
Source: eLife. 2023 Apr 3;12:e83217. doi: 10.7554/eLife.83217 (PMC10069869; doi:10.7554/eLife.83217)
Supplement: Figure 2—source data 12. [file elife-83217-fig2-data12.pdf]

ABE on deep layer primary cortical neurons

3rd ABE 5-12-11
Exposure: ECL 30″

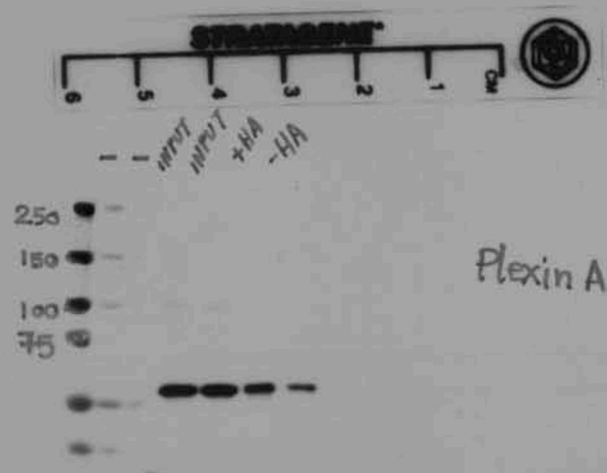

Plexin A3 Ab, rabbit (AbCam)

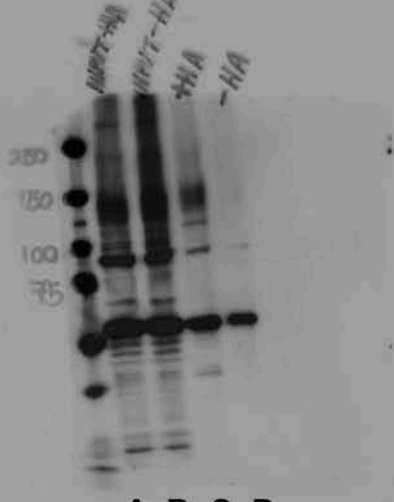

A B C D

A: Input +HA
B: Input -HA
C: +HA
D: -HA

Nrp-1 immunoblot

Nrp1 IB: α-Nrp1 Ab, goat (RandD)
1:1000 in 3% BSA/TBS-T overnight at 4°C

2°: α-goat HRP-conjugated Ab
1:10000 Text 1% milk

16 μl/lane from [120 μl sample + 40 μl 4X Laemmli]
