# Supplementary material for: Palmitoylation regulates neuropilin-2 localization and function in cortical neurons and conveys specificity to semaphorin signaling via palmitoyl acyltransferases
Source: eLife. 2023 Apr 3;12:e83217. doi: 10.7554/eLife.83217 (PMC10069869; doi:10.7554/eLife.83217)

ABE on Neuro2A cells expressing Nrp-2 plasmids

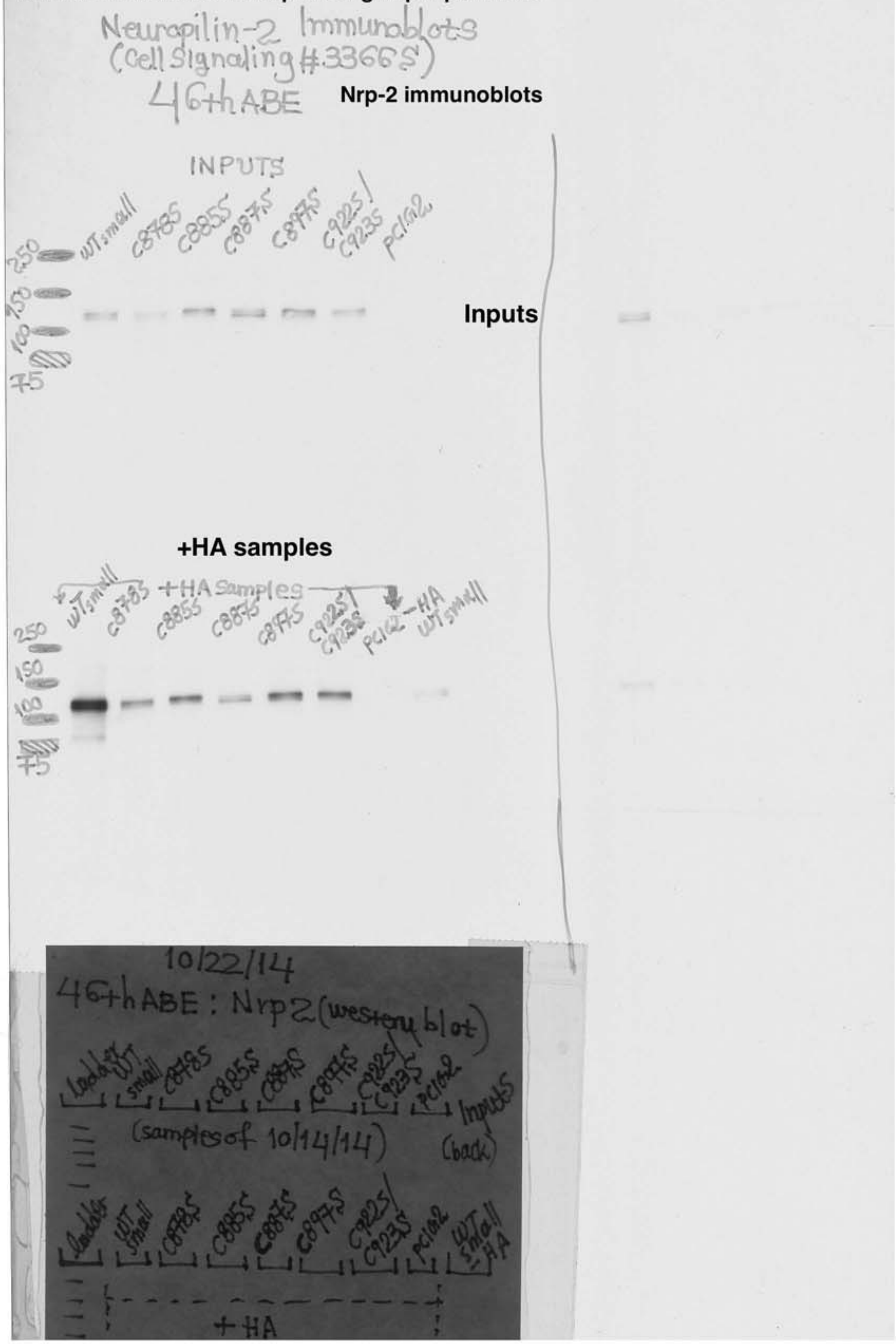

Supplement: Figure 2—source data 16. [file elife-83217-fig2-data16.pdf]
