# Supplementary material for: Palmitoylation regulates neuropilin-2 localization and function in cortical neurons and conveys specificity to semaphorin signaling via palmitoyl acyltransferases
Source: eLife. 2023 Apr 3;12:e83217. doi: 10.7554/eLife.83217 (PMC10069869; doi:10.7554/eLife.83217)
Supplement: Figure 2—source data 20. [file elife-83217-fig2-data20.pdf]

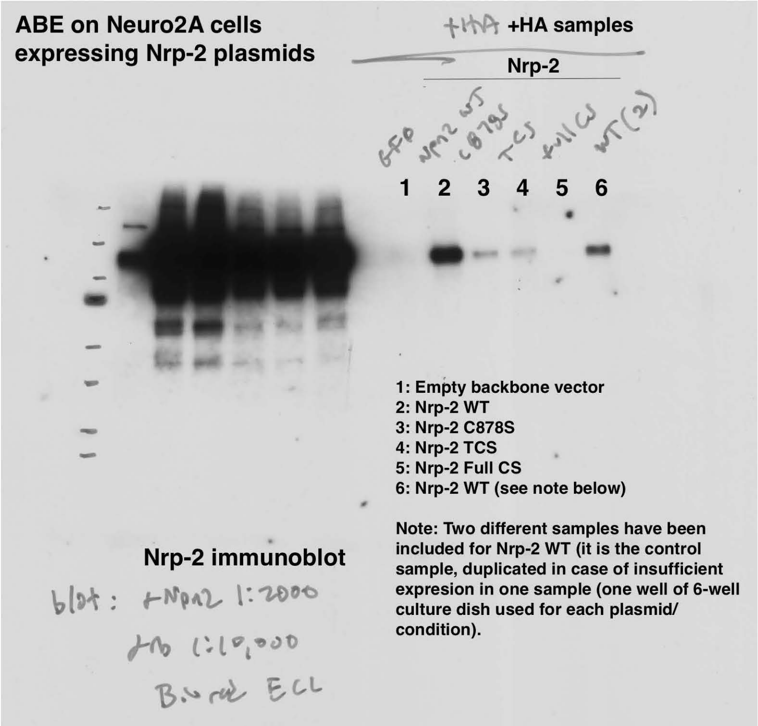
ABE on Neuro2A cells
expressing Nrp-2 plasmids
+HA samples
Nrp-2
GFP
Nrp2 WT
C878S
TCS
full CS
WT (2)
1 2 3 4 5 6
1: Empty backbone vector
2: Nrp-2 WT
3: Nrp-2 C878S
4: Nrp-2 TCS
5: Nrp-2 Full CS
6: Nrp-2 WT (see note below)
Note: Two different samples have been included for Nrp-2 WT (it is the control sample, duplicated in case of insufficient expresion in one sample (one well of 6-well culture dish used for each plasmid/ condition).
Nrp-2 immunoblot
blot:
1:2000
