# Supplementary material for: Palmitoylation regulates neuropilin-2 localization and function in cortical neurons and conveys specificity to semaphorin signaling via palmitoyl acyltransferases
Source: eLife. 2023 Apr 3;12:e83217. doi: 10.7554/eLife.83217 (PMC10069869; doi:10.7554/eLife.83217)
Supplement: Figure 2—source data 21. [file elife-83217-fig2-data21.pdf]

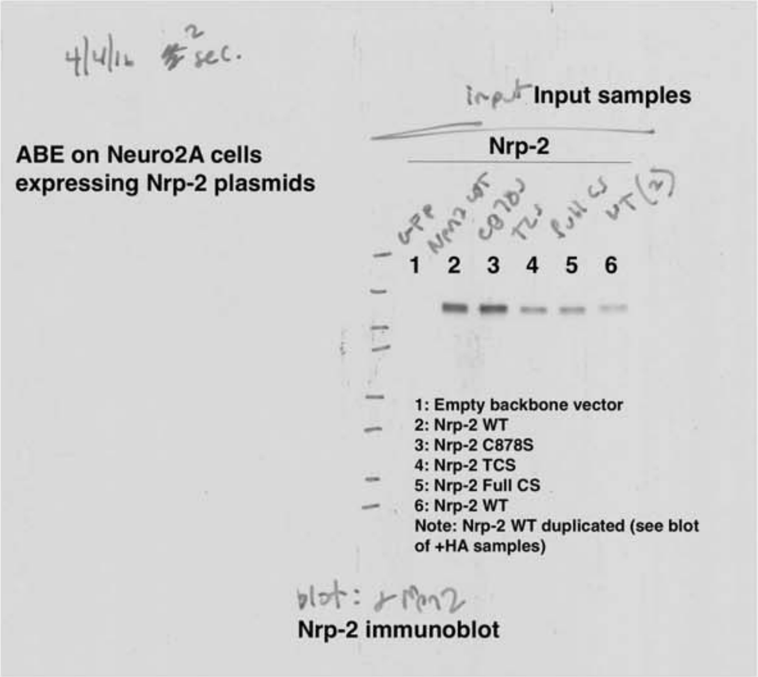

Input samples
Nrp-2
ABE on Neuro2A cells expressing Nrp-2 plasmids
1 2 3 4 5 6
1: Empty backbone vector
2: Nrp-2 WT
3: Nrp-2 C878S
4: Nrp-2 TCS
5: Nrp-2 Full CS
6: Nrp-2 WT
Note: Nrp-2 WT duplicated (see blot of +HA samples)
Nrp-2 immunoblot
