# Supplementary material for: Palmitoylation regulates neuropilin-2 localization and function in cortical neurons and conveys specificity to semaphorin signaling via palmitoyl acyltransferases
Source: eLife. 2023 Apr 3;12:e83217. doi: 10.7554/eLife.83217 (PMC10069869; doi:10.7554/eLife.83217)
Supplement: Figure 3—figure supplement 3—source data 3. [file elife-83217-fig3-figsupp3-data3.pdf]

Golgi isolation from mouse whole brain

Six fractions (shown below) were collected from Golgi preparations

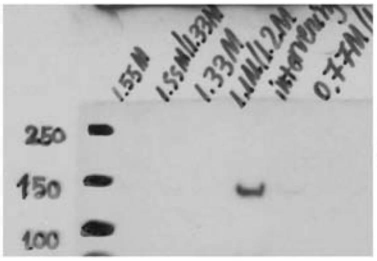

GM130 immunoblot (identifies the cis-Golgi fraction)
