# Supplementary material for: Palmitoylation regulates neuropilin-2 localization and function in cortical neurons and conveys specificity to semaphorin signaling via palmitoyl acyltransferases
Source: eLife. 2023 Apr 3;12:e83217. doi: 10.7554/eLife.83217 (PMC10069869; doi:10.7554/eLife.83217)
Supplement: Figure 3—figure supplement 4—source data 5. [file elife-83217-fig3-figsupp4-data5.pdf]

Myc immunoblot (PlexA3)
IP: Nrp-2

Input **CoIP**

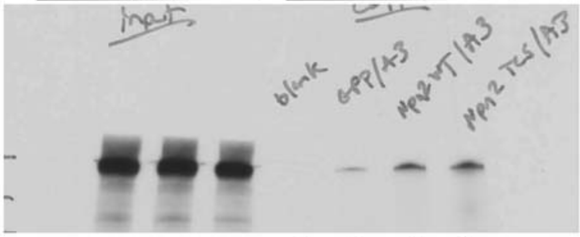

1 2 3 4

1: Backbone vector
2: Backbone vector + Myc-PlexA3
3: Flag-Nrp-2 WT + Myc-PlexA3
4: Flag-Nrp-2 Full CS + Myc-PlexA3 (*on the blot this lane is mislabeled: labeled TCS instead of the correct Full CS)
