# Supplementary material for: Palmitoylation regulates neuropilin-2 localization and function in cortical neurons and conveys specificity to semaphorin signaling via palmitoyl acyltransferases
Source: eLife. 2023 Apr 3;12:e83217. doi: 10.7554/eLife.83217 (PMC10069869; doi:10.7554/eLife.83217)
Supplement: Figure 3—figure supplement 4—source data 6. [file elife-83217-fig3-figsupp4-data6.pdf]

Myc immunoblot (PlexA3)

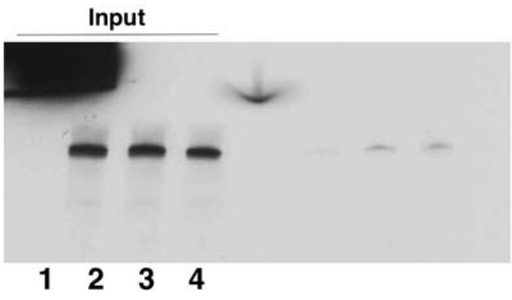

1: Backbone vector
2: Backbone vector + Myc-PlexA3
3: Flag-Nrp-2 WT + Myc-PlexA3
4: Flag-Nrp-2 Full CS + Myc-PlexA3
