# Supplementary material for: Palmitoylation regulates neuropilin-2 localization and function in cortical neurons and conveys specificity to semaphorin signaling via palmitoyl acyltransferases
Source: eLife. 2023 Apr 3;12:e83217. doi: 10.7554/eLife.83217 (PMC10069869; doi:10.7554/eLife.83217)
Supplement: Figure 3—figure supplement 4—source data 8. [file elife-83217-fig3-figsupp4-data8.pdf]

## Nrp-2 immunoblot

**Input**

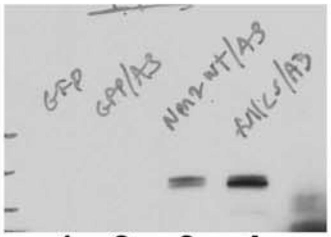


**1 2 3 4**

**1: Backbone vector (empty)**
**2: Backbone vector + Myc-PlexA3**
**3: Flag-Nrp-2 WT + Myc-PlexA3**
**4: Flag-Nrp-2 Full CS + Myc-PlexA3**
