# Supplementary material for: Palmitoylation regulates neuropilin-2 localization and function in cortical neurons and conveys specificity to semaphorin signaling via palmitoyl acyltransferases
Source: eLife. 2023 Apr 3;12:e83217. doi: 10.7554/eLife.83217 (PMC10069869; doi:10.7554/eLife.83217)
Supplement: Figure 3—figure supplement 4—source data 20. [file elife-83217-fig3-figsupp4-data20.pdf]

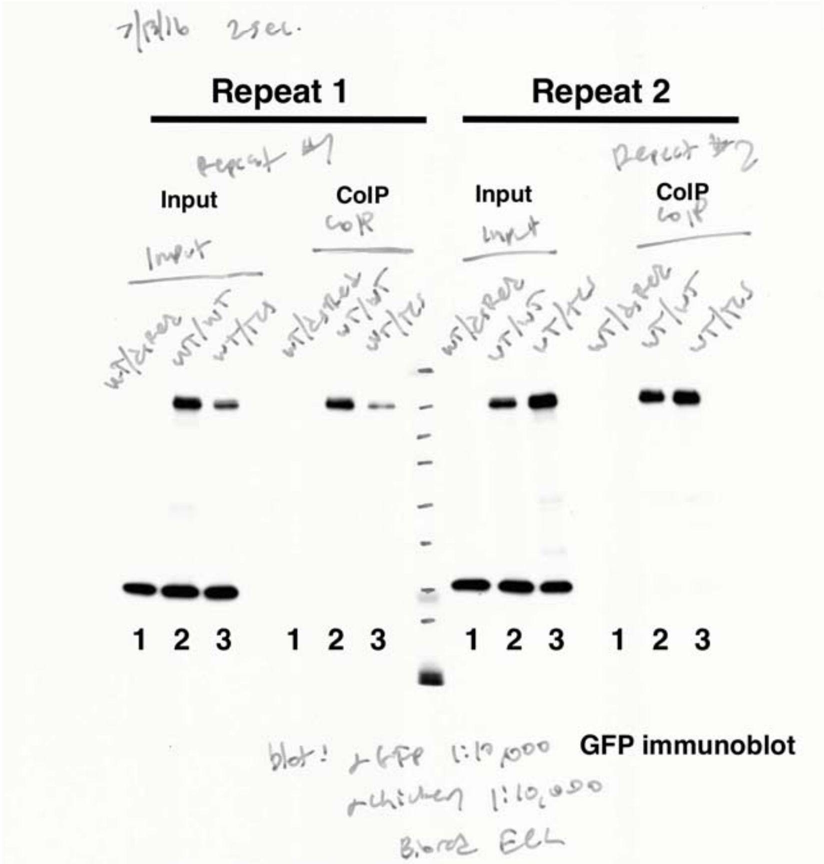

1: Flag-Nrp-2 WT + Backbone vector
2: Flag-Nrp-2 WT + pHluorin-Nrp-2 WT
3: Flag-Nrp-2 WT + pHluorin-Nrp-2 TCS
