# Supplementary material for: Palmitoylation regulates neuropilin-2 localization and function in cortical neurons and conveys specificity to semaphorin signaling via palmitoyl acyltransferases
Source: eLife. 2023 Apr 3;12:e83217. doi: 10.7554/eLife.83217 (PMC10069869; doi:10.7554/eLife.83217)
Supplement: Figure 4—source data 2. [file elife-83217-fig4-data2.pdf]

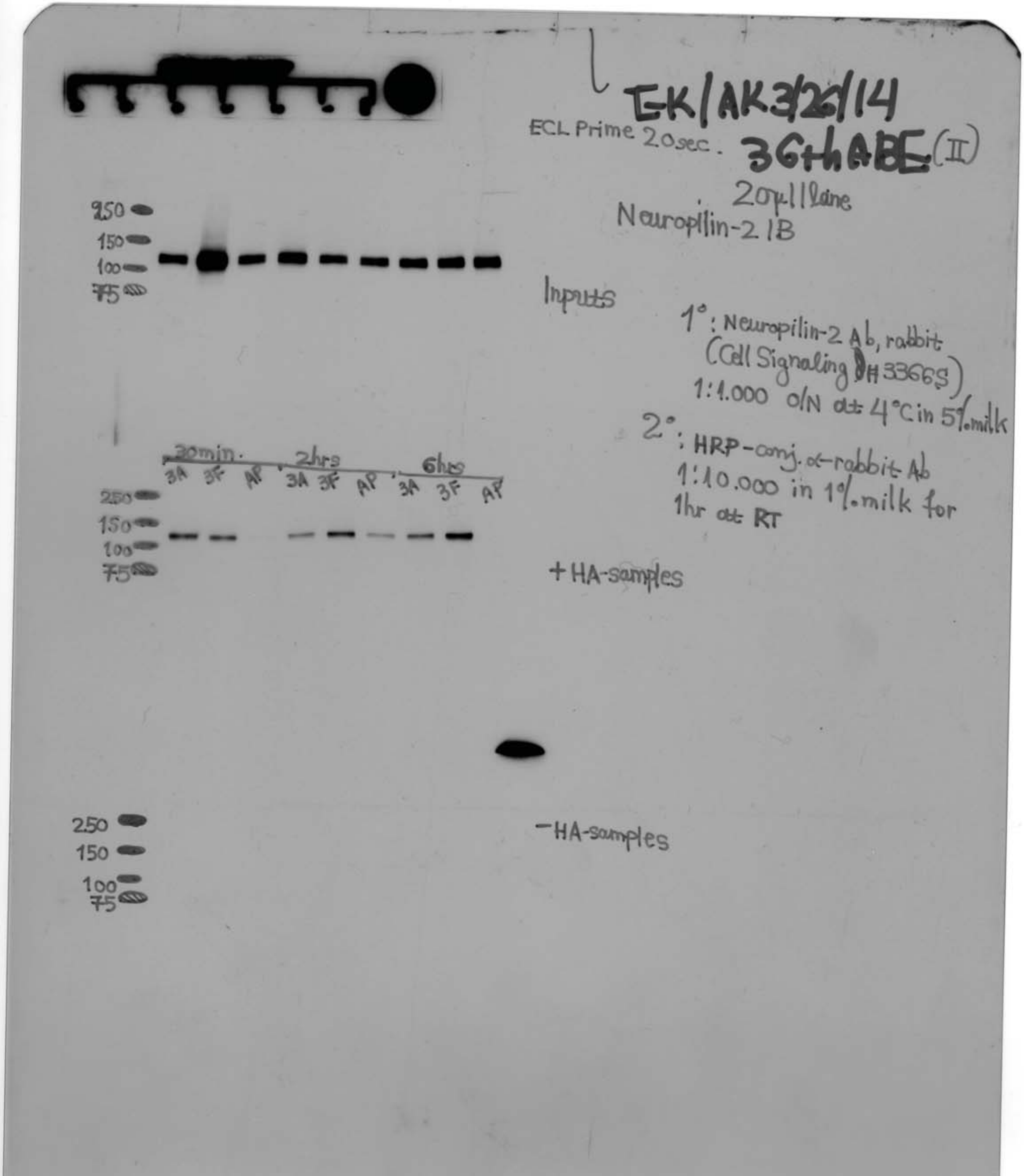
EK/AK 3/26/14
ECL Prime 20sec.
36th ABE (II)
20µl/lane
Neuropilin-2 IB
250
150
100
75
Inputs
1°: Neuropilin-2 Ab, rabbit
(Cell Signaling #3366S)
1:1.000 O/N at 4°C in 5% milk
2°: HRP-conj. α-rabbit Ab
1:10.000 in 1% milk for
1hr at RT
30min.
2hrs
6hrs
3A
3F
AP
+HA-samples
-HA-samples
