# Supplementary material for: Palmitoylation regulates neuropilin-2 localization and function in cortical neurons and conveys specificity to semaphorin signaling via palmitoyl acyltransferases
Source: eLife. 2023 Apr 3;12:e83217. doi: 10.7554/eLife.83217 (PMC10069869; doi:10.7554/eLife.83217)
Supplement: Figure 4—source data 3. [file elife-83217-fig4-data3.pdf]

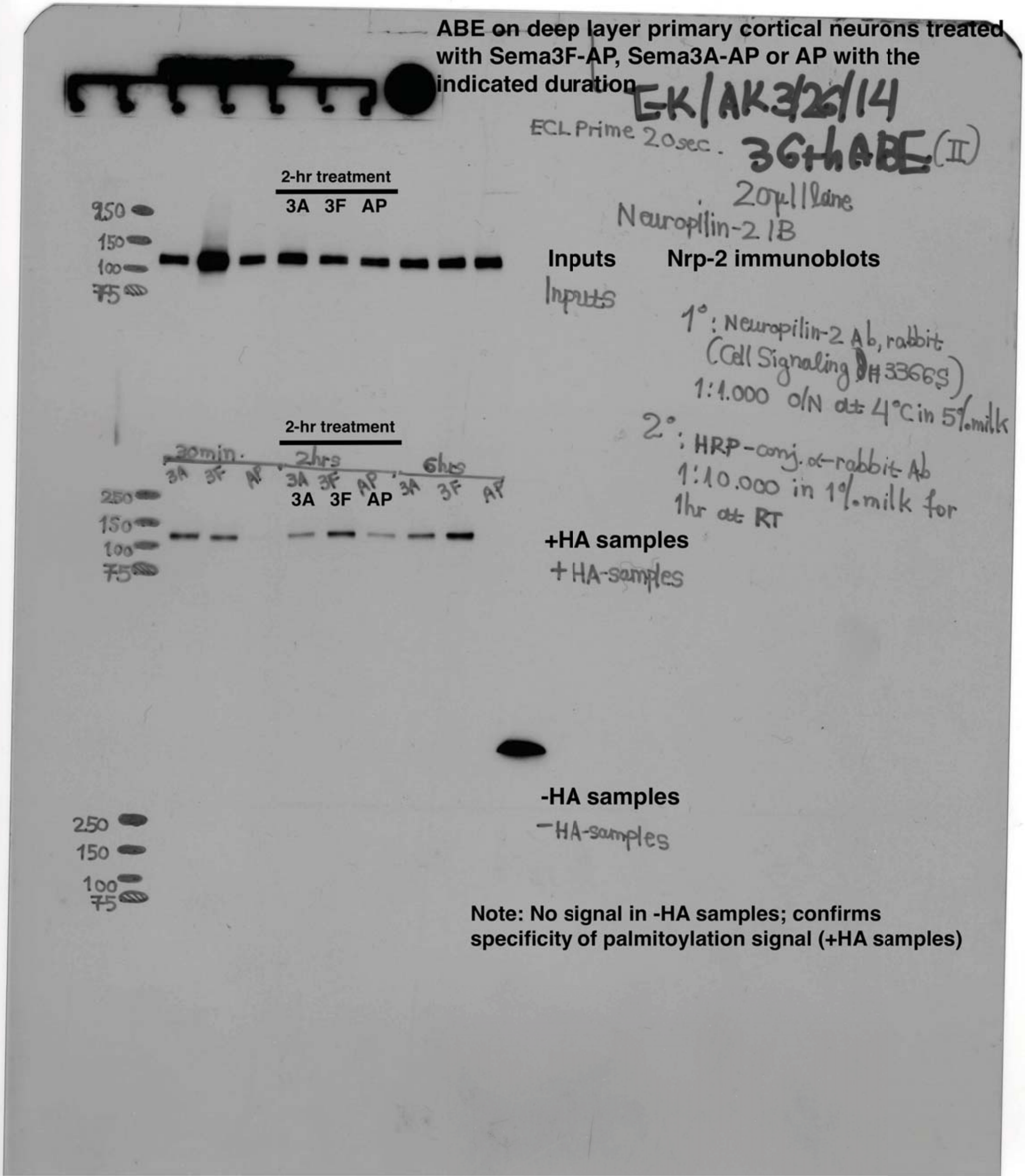
ABE on deep layer primary cortical neurons treated with Sema3F-AP, Sema3A-AP or AP with the indicated duration
EK/AK 3/26/14
ECL Prime 20sec.
36th ABE (II)
20µl/lane
Neuropilin-2 IB
2-hr treatment
3A 3F AP
250
150
100
75
Inputs
Nrp-2 immunoblots
Inputs
1°: Neuropilin-2 Ab, rabbit (Cell Signaling #3366S)
1:1.000 O/N at 4°C in 5% milk
2°: HRP-conj. α-rabbit Ab
1:10.000 in 1% milk for 1hr at RT
2-hr treatment
30min. 2hrs 6hrs
3A 3F AP 3A 3F AP 3A 3F AP
3A 3F AP
+HA samples
+HA-samples
-HA samples
-HA-samples
Note: No signal in -HA samples; confirms specificity of palmitoylation signal (+HA samples)
