# Supplementary material for: Palmitoylation regulates neuropilin-2 localization and function in cortical neurons and conveys specificity to semaphorin signaling via palmitoyl acyltransferases
Source: eLife. 2023 Apr 3;12:e83217. doi: 10.7554/eLife.83217 (PMC10069869; doi:10.7554/eLife.83217)
Supplement: Figure 6—source data 2. [file elife-83217-fig6-data2.pdf]

GK/AK
12/2/14

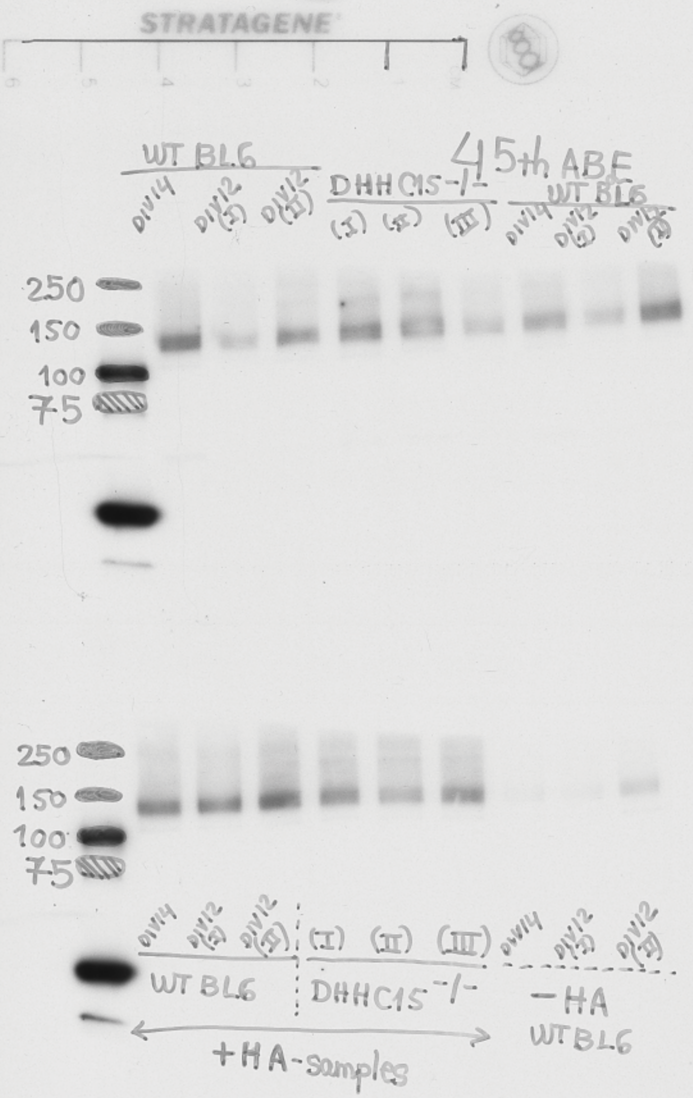

ECL Prime 3''

INPUTS

IB: Neuropilin-1 Ab, rabbit 1:1.000
(abCam #ab 81321)
o/N at 4°C
2°: α-rb HRP-conjugated Ab
1:10.000 for 1hr at RT

processed Samples

20μl/lane

12/1/14 45th ABE
Neuropilin-1 WB
ladder DIV14 DIV12 (I) DIV12 (II) I II III WT BL6 DIV14 DIV12 (I) DIV12 (II)
WT BL6 | DHHC15 -/- | -HA
+HA
20μl/lane
Samples of: 10/8/14 & 10/11/14
Same order for Inputs and processed samples
1°Ab: ab81321 rb mAb to Neuropilin1 [EPR3113]
