# Supplementary material for: Palmitoylation regulates neuropilin-2 localization and function in cortical neurons and conveys specificity to semaphorin signaling via palmitoyl acyltransferases
Source: eLife. 2023 Apr 3;12:e83217. doi: 10.7554/eLife.83217 (PMC10069869; doi:10.7554/eLife.83217)
Supplement: Figure 6—figure supplement 1—source data 1. [file elife-83217-fig6-figsupp1-data1.pdf]

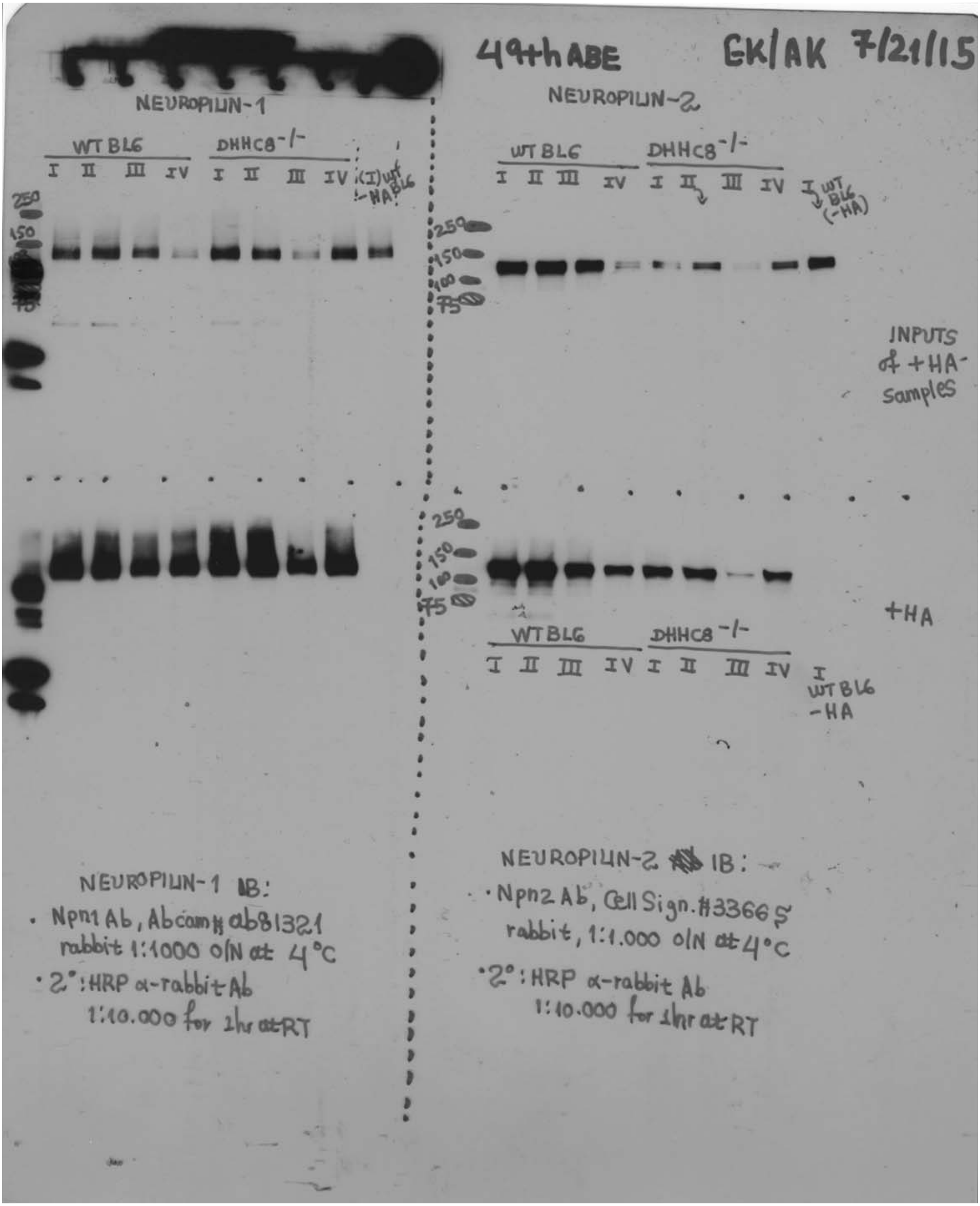
49th ABE
EK/AK 7/21/15
NEUROPILIN-1
WT BL6
DHHC8 -/-
I II III IV I II III IV (I) WT BL6 -HA
250
150
NEUROPILIN-2
WT BL6
DHHC8 -/-
I II III IV I II III IV I WT BL6 (-HA)
250
150
100
75
INPUTS of +HA-samples
250
150
100
75
+HA
WT BL6
DHHC8 -/-
I II III IV I II III IV
I WT BL6 -HA
NEUROPILIN-1 IB:
• Npn1 Ab, Abcam# ab81321 rabbit 1:1000 O/N at 4°C
• 2°: HRP α-rabbit Ab 1:10.000 for 1hr at RT
NEUROPILIN-2 IB:
• Npn2 Ab, Cell Sign. #3366S rabbit, 1:1.000 O/N at 4°C
• 2°: HRP α-rabbit Ab 1:10.000 for 1hr at RT
