# Supplementary material for: Palmitoylation regulates neuropilin-2 localization and function in cortical neurons and conveys specificity to semaphorin signaling via palmitoyl acyltransferases
Source: eLife. 2023 Apr 3;12:e83217. doi: 10.7554/eLife.83217 (PMC10069869; doi:10.7554/eLife.83217)
Supplement: Figure 6—figure supplement 1—source data 2. [file elife-83217-fig6-figsupp1-data2.pdf]

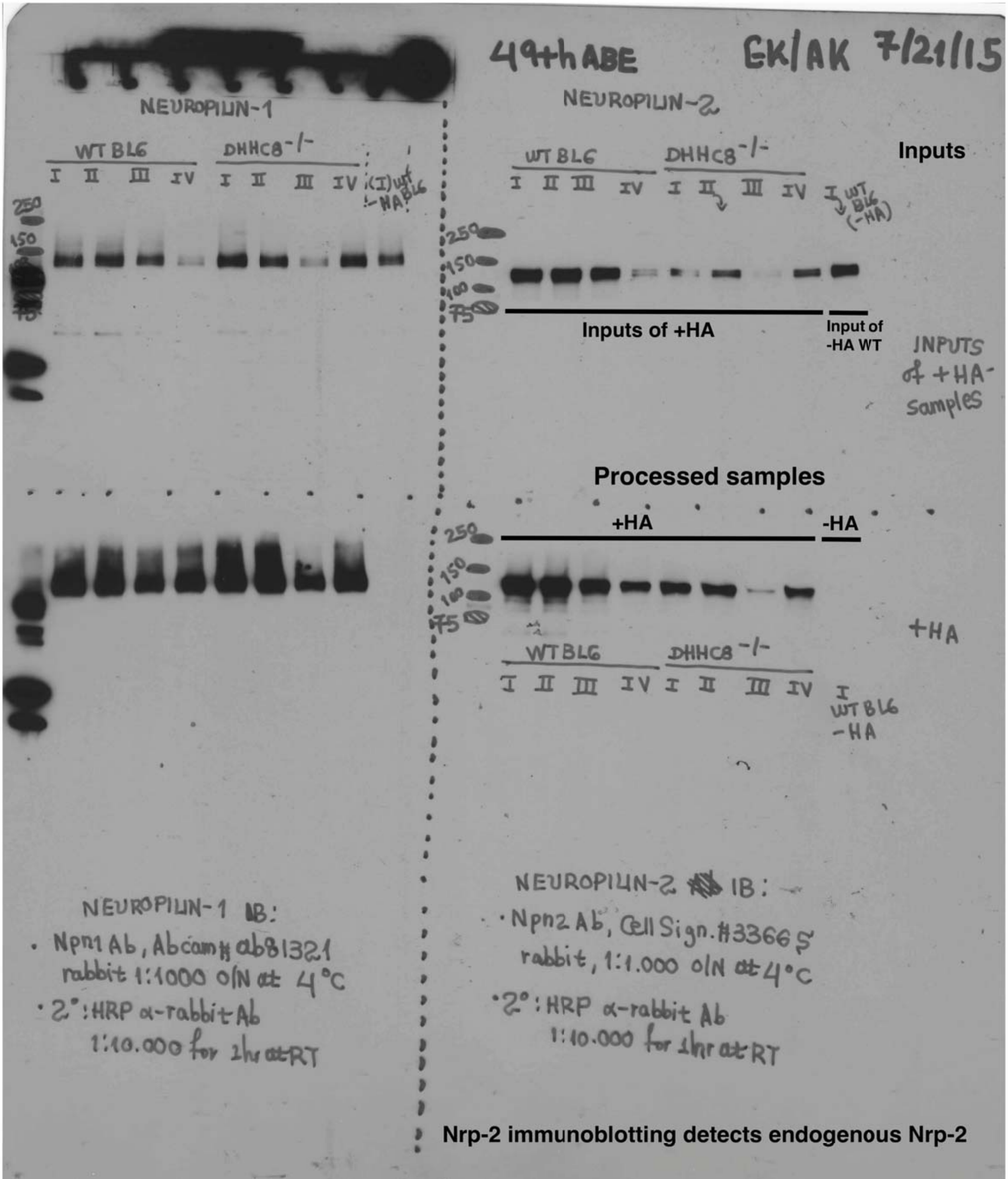

ABE on WT or DHHC8-/- deep layer primary cortical neurons
