# Supplementary material for: Palmitoylation regulates neuropilin-2 localization and function in cortical neurons and conveys specificity to semaphorin signaling via palmitoyl acyltransferases
Source: eLife. 2023 Apr 3;12:e83217. doi: 10.7554/eLife.83217 (PMC10069869; doi:10.7554/eLife.83217)

EK/AK
10/23/14

ABE on WT or DHHC15-/- deep layer primary cortical neurons
Nrp-2 immunoblots detect endogenous Nrp-2

10µl/lane (all samples)

45th ABE

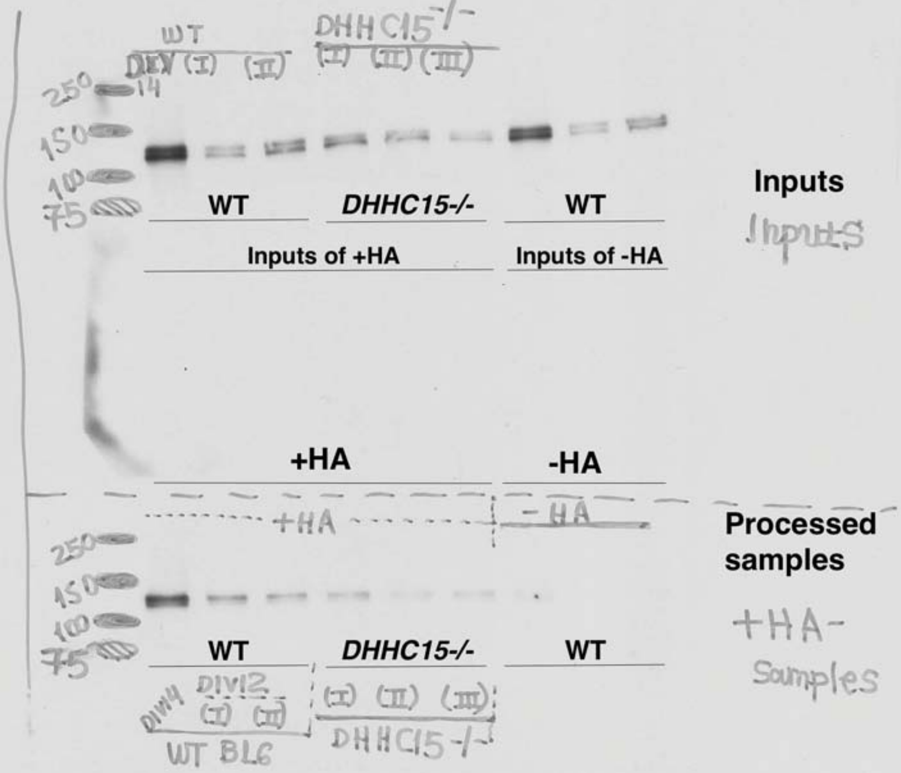

Neuropilin-2 1B:
Nrp2 Ab 1:1.000
(Cell Sign.#3366S)
O/N at 4°C
2°: HRP-conjug.
α-rabbit Ab
1:10.000
1hr at RT

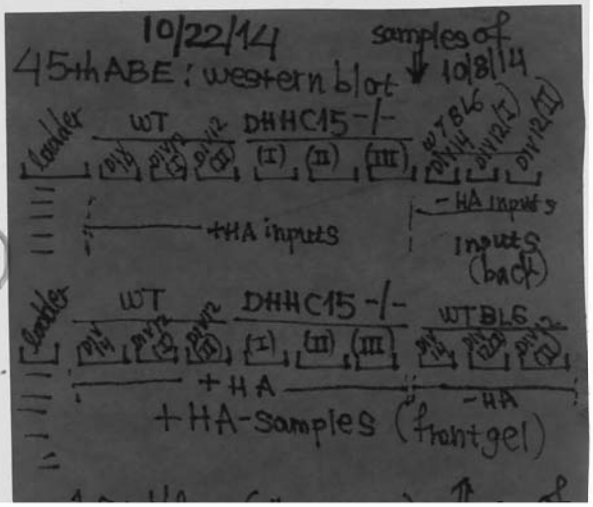

Supplement: Figure 6—source data 3. [file elife-83217-fig6-data3.pdf]
